# Extra-skeletal manifestations in mice affected by *Clcn7*-dependent autosomal dominant osteopetrosis type 2 clinical and therapeutic implications

**DOI:** 10.1038/s41413-019-0055-x

**Published:** 2019-06-11

**Authors:** Antonio Maurizi, Mattia Capulli, Annabel Curle, Rajvi Patel, Argia Ucci, Juliana Alves Côrtes, Harriet Oxford, Shireen R. Lamandé, John F. Bateman, Nadia Rucci, Anna Teti

**Affiliations:** 1 Department of Biotechnological and Applied Clinical Sciences, University of L’Aquila, L’Aquila, Italy; 2 Murdoch Children’s Research Institute and University of Melbourne, Melbourne, Australia

**Keywords:** Pathogenesis, Diseases

## Abstract

Autosomal dominant osteopetrosis type 2 (ADO2) is a high-density brittle bone disease characterized by bone pain, multiple fractures and skeletal-related events, including nerve compression syndrome and hematological failure. We demonstrated that in mice carrying the heterozygous *Clcn7*^*G213R*^ mutation, whose human mutant homolog *CLCN7*^*G215R*^ affects patients, the clinical impacts of ADO2 extend beyond the skeleton, affecting several other organs. The hallmark of the extra-skeletal alterations is a consistent perivascular fibrosis, associated with high numbers of macrophages and lymphoid infiltrates. Fragmented clinical information in a small cohort of patients confirms extra-skeletal alterations consistent with a systemic disease, in line with the observation that the *CLCN7* gene is expressed in many organs. ADO2 mice also show anxiety and depression and their brains exhibit not only perivascular fibrosis but also β-amyloid accumulation and astrogliosis, suggesting the involvement of the nervous system in the pathogenesis of the ADO2 extra-skeletal alterations. Extra-skeletal organs share a similar cellular pathology, confirmed also in vitro in bone marrow mononuclear cells and osteoclasts, characterized by an impairment of the exit pathway of the *Clcn7* protein product, ClC7, through the Golgi, with consequent reduced ClC7 expression in late endosomes and lysosomes, associated with high vesicular pH and accumulation of autophagosome markers. Finally, an experimental siRNA therapy, previously proven to counteract the bone phenotype, also improves the extra-skeletal alterations. These results could have important clinical implications, supporting the notion that a systematic evaluation of ADO2 patients for extra-skeletal symptoms could help improve their diagnosis, clinical management, and therapeutic options.

## Introduction

Autosomal dominant osteopetrosis type 2 (ADO2) is a rare disease known to affect the skeleton.^1^ First recognized by Albers-Schönberg^2^ and called Albers-Schönberg disease or marble bone disease,^3^ ADO2 is now documented to affect 1 in 20 000 live births.^4,5^ It has an autosomal dominant inheritance, with about 70% of patients carrying heterozygous missense mutations of the *CLCN7* gene encoding the ClC7 2Cl^−^/1H^+^ antiporter.^6–8^ This antiporter is intrinsic to the acidic organelles, including lysosomes. In osteoclasts, it also localizes in the so-called ruffled border, a convoluted plasma membrane domain facing the bone surface during the process of bone resorption that protrudes into the resorption lacuna, representing the extracellular environment between the bone surface and the osteoclast membrane.^9^ Osteoclasts are the cells thought to be primarily affected by *CLCN7* mutations as they rely on this co-transporter to charge balance both acidic organelles and resorption lacuna.^9^ Impairment of resorption lacuna acidification blocks bone resorption and prevents matrix renewal, making the bones dense but fragile.^10^ ADO2 patients are heterozygous and, although they have a less severe phenotype than homozygous patients,^1,11^ they still suffer from severe pain, multiple fractures that are difficult to heal, nerve compression syndromes, and hematological failures, probably due to nerve foramina and medullary cavity constraints, respectively.^1,11,12^ In rare cases, mutations are life-threatening.^4,5,13,14^

Given the prevalent skeletal complications, ADO2 patients are not evaluated systematically for extra-skeletal manifestations and data on the involvement of other organs in ADO2 are scant and fragmented. However, some extra-skeletal implications can be assumed from the consistent increase of creatine kinase in several patients,^15^ which could reflect an underestimated myopathy.

An important limitation in the full understanding of *CLCN7*-dependent ADO2 pathophysiology until recently was the lack of a genuine ADO2 mouse model. This was addressed in 2014 by the generation of an ADO2 mouse carrying the murine homolog (*Clcn7*^*G213R*^) of the most frequent human ADO2 mutation (*CLCN7*^*G215R*^).^7,16^ Heterozygous *Clcn7*^*G213R*^ mice manifest skeletal alterations like patients and have been extensively investigated for their bone phenotype^16^ and to test therapies.^17–19^ Review of the literature and our systematic evaluation of wild-type (WT) mice demonstrated that *Clcn7* is expressed in many organs and cell types beyond osteoclasts,^6,20,21^ prompting us to hypothesize that *CLCN7*-dependent ADO2 could present with important extra-skeletal complications, which could contribute to morbidity. We confirmed this hypothesis showing alterations in several organs of ADO2 mice, possibly explaining some fragmented information available in our small cohort of patients.^14^ Our observations may have important clinical implications, opening the door to a more comprehensive and systematic evaluation of ADO2 patients, who could greatly benefit from an accurate examination of the skeletal and also the extra-skeletal phenotype. Furthermore, we demonstrated that extra-skeletal manifestations are normalized by treatment with a specific siRNA therapy previously proven by our group to be safe and effective on the ADO2 mouse bone phenotype^17,19^ and on in vitro bone resorption by osteoclasts differentiated from the peripheral blood mononuclear cells of *a CLCN7*^*G215R*^ ADO2 patient.^17^

## Results

### Visceral and somatic phenotype

**Fig. 1:**
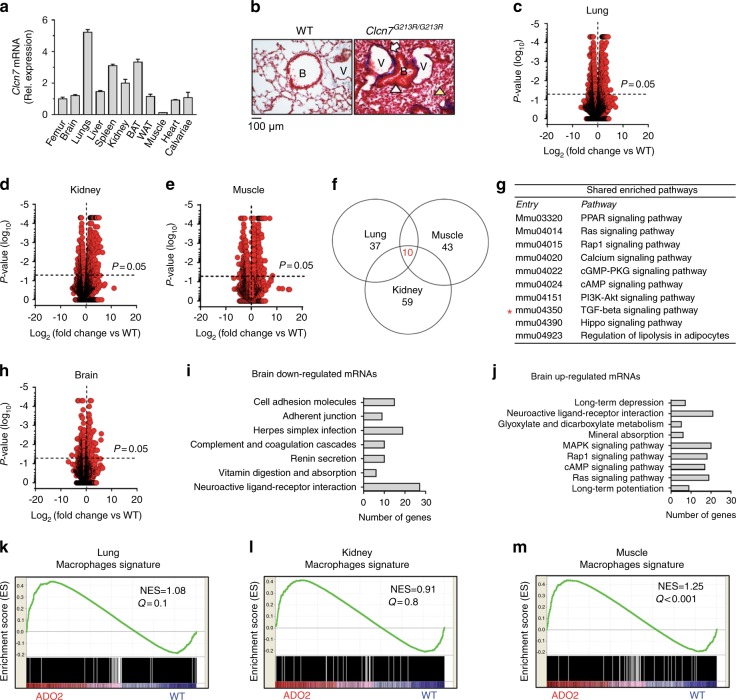
Extra-skeletal pathways in ADO2 patients and mice. **a** Real-time RT-PCR for *Clcn7* in the indicated organs of 3-month-old wild-type (WT) C57BL6/J male mice. **b** Histological sections of WT and homozygous *Clcn7*^*G213R/G213R*^ mouse lungs (Masson’s trichrome staining). White arrowhead: closed airway. Yellow arrowhead: alveolar atelectasis. V: vessel. B: bronchiole. **c** RNA-dSeq analysis in lungs, kidneys muscles, and brains from 1-month-old WT and ADO2 C57BL6/J male mice. RNA-dSeq and Volcano plot of differential gene expression in ADO2 lungs, **d** kidneys and **e** muscle. Horizontal dashed red line indicates the *P*-value threshold. **f** Venn diagram of the statistically significant enriched pathways. Red: number of enriched pathways shared by ADO2 lungs, muscles and kidneys. **g** List of the shared enriched pathways in the ADO2 organs. **h** RNA-dSeq and Volcano plot of differential gene expression in ADO2 brains. **i** Statistically significant pathways enriched in the set of genes under-expressed and **j** over-expressed in ADO2 vs. WT brain. **k** Macrophages gene signature in ADO2 lungs, **l** kidneys and **m** muscles. NES normalized enrichment score. Images are representative, and data are the mean ± S.D. of three mice per organ

Data from a group of 21 ADO2 patients^14^ were available in our laboratory. Eight of these patients received a genetic diagnosis of *CLCN7* mutations and were re-examined, in this study, for any clinical manifestations consistent with an extra-skeletal disease. Besides the typical radiological manifestations of ADO2,^14^ we noted that these patients could also present cerebral, hematological, renal and splenic alterations (Table 1). While hematological alterations affected most patients and could be explained by the medullary constraints generated by the lack of bone resorption, and hence directly related to the skeletal phenotype, the other manifestations appeared independent of osteopetrosis and could imply an organ-autonomous disease. This is in accordance with the observation that several organs express the *CLCN7/Clcn7* gene in humans and mice,^6,20,21^ as confirmed in this study in Fig. 1a, and in Supplemental Fig. 1a–h in which we normalized the *Clcn7* expression with the housekeeping genes, *Gapdh*, *Hprt*, *and β-actin*. The lowest *Clcn7* expression was found in the muscle, while the lung showed the highest *Clcn7* level, with a >5-fold greater expression than in bone (Fig. 1a and Supplemental Fig. 1a). According to the high lung *Clcn7* expression, severe alveolar atelectasis and air way closure was observed in the lung of lethal homozygous mutant mice (Fig. 1b). So far, this observation has not been reported in literature for humans, but it could be of clinical relevance by contributing to the high morbidity typical of homozygous patients carrying *CLCN7* loss-of-function mutations.^11,22^

**Table 1:** Extra-skeletal alterations in ADO2 patients

Age	(13–60) y
Gender	Male 50% (4) female 50% (4)
Genetic diagnosis of Clcn7 mutation	100% (8/8)
Neurological alterations	
EEG abnormalities in the frontotemporal cortex	12.5% (1/8)
Epilepsy, ipsaritmia and speech disorder	12.5% (1/8)
Anemia	62.5% (6/8)
Leucopenia	12.5% (1/8)
Muscle alterations	
Myopathy (altered electromyogram)	12.5% (1/8)
High serum creatine kinase	25% (2/8)
Kidney alterations	
High creatininemia and low creatinine clearence	25% (2/8)
Splenic alteration	
Splenomegaly	12.5% (1/8)

Although the clinical evidence shown in Table 1 had no power due to the small size of the cohort, it prompted us to investigate the molecular changes induced by the *Clcn7*^*G213R*^ mutation in ADO2 mouse extra-skeletal organs. Therefore, we isolated the total RNA from WT and ADO2 lungs, kidneys and muscles, as examples of organs exhibiting high, medium, and low *Clcn7* expression. The total RNAs isolated from five mice/group were pooled and analyzed by RNA deep sequencing (RNA-dSeq). To our surprise, not only lungs and kidneys, but also muscles showed differential transcriptome profile in ADO2 compared to WT organs (Fig. 1c–e). Modulated genes were grouped, and pathway enrichment was evaluated for each group of organs. Interestingly, ten enriched molecular pathways were shared by the three ADO2 organs (Fig. 1f, g), including the TGFβ signaling, highlighted in Fig. 1g (asterisk) for reasons explained below. We also observed that several molecular pathways were altered in the ADO2 brains (Fig. 1h), including both downregulated (Fig. 1i) and upregulated (Fig. 1j) genes, prompting us to hypothesize a behavioral and/or cognitive phenotype in ADO2 mice.

Using gene set enrichment analysis (GSEA) software we were also able to compare our RNA-dSeq database with database containing specific gene signatures (Supplemental Table 1). The analysis showed that lungs, kidneys, and muscles shared a macrophage enriched gene signature in ADO2 compared to WT organs (Fig. 1k–m). Taken together, these results indicate that at least four extra-skeletal organs are affected by the *Clcn7*^*G213R*^ mutation, including the low-*Clcn7* expressing muscle, and that at least part of the molecular alterations induced by the mutant gene could be associated with an enrichment of macrophages.

**Fig. 2:**
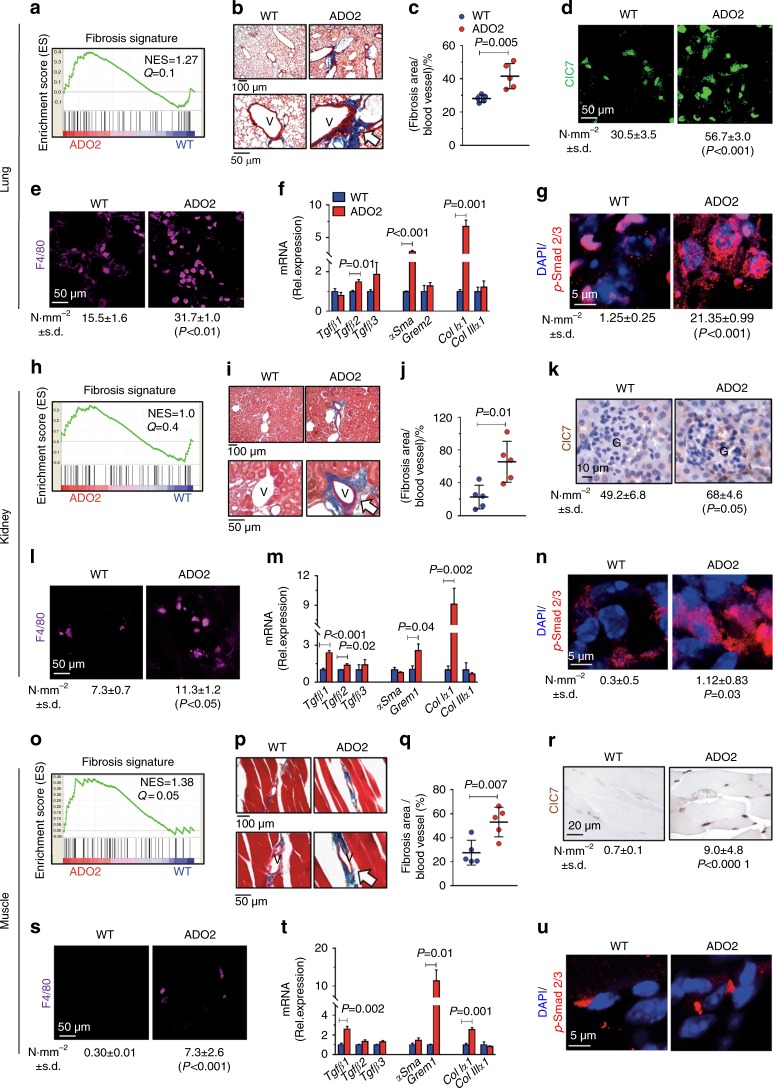
Lung, kidney, and muscle phenotype. Lungs, kidneys, and muscles were harvested from 12-month-old WT and ADO2 CD1 male mice. **a** Analysis of fibrosis gene signature in RNA-dSeq data sets of 1-month-old WT and ADO2 C57BL6/J male mice lungs. NES normalized enrichment score. **b** Masson’s trichrome staining of WT and ADO2 lung sections. White arrows: perivascular collagen (blue) accumulation. V: vessel. **c** Quantification of perivascular collagen. **d** Immunofluorescence analyses of ClC7 (green) and **e** the macrophage marker F4/80 (purple) in WT and ADO2 lung sections. N·mm^−2^, number per mm^2^. **f** Real time RT-PCR for the indicated genes in WT and ADO2 lungs. **g** Immunofluorescence analysis of phospho (p)-Smad2/3 (red) in WT and ADO2 lung sections. **h** Enrichment plots for fibrosis gene signature in ADO2 kidneys. NES normalized enrichment score. **i** Masson’s trichrome staining of WT and ADO2 kidney sections. White arrows: perivascular collagen (blue) accumulation. V: vessel. **j** Quantification of perivascular collagen. **k** Immunohistochemistry for ClC7 (brown) in WT and ADO2 kidney sections. **l** Immunofluorescence analysis of the macrophage marker F4/80 (purple) in WT and ADO2 kidney sections. **m** Real time RT-PCR for the indicated genes in WT and ADO2 kidney*s*. **n** Immunofluorescence analysis of p-Smad2/3 (red) in WT and ADO2 kidney sections. **o** Enrichment plots of fibrosis gene signature in ADO2 muscles. NES normalized enrichment score. **p** Masson’s trichrome staining of WT and ADO2 muscle sections. White arrows: perivascular collagen (blue) accumulation. V: vessel. **q** Quantification of perivascular collagen. **r** Immunohistochemistry for ClC7 (brown) in WT and ADO2 muscle sections. **s** Immunofluorescence analysis for the macrophage marker F4/80 (purple) in WT and ADO2 muscle sections. **t** Real time RT-PCR for the indicated genes in WT and ADO2 muscles. **u** Immunofluorescence analysis of p-Smad1/2 (red) in WT and ADO2 muscle sections. Nuclei are stained in blue with DAPI. Images are representative, and data are the mean ± S.D of five mice per group (Student’s *t* test)

Our bioinformatic analysis, performed by the GSEA software, also revealed an enrichment of the fibrotic signature in ADO2 lungs (Fig. 2a). Therefore, we performed a Masson’s trichrome staining to unveil collagen fibers and observed perivascular fibrosis in ADO2 compared to WT lungs (Fig. 2b, c). Furthermore, an immunofluorescence analysis demonstrated that more cells expressed ClC7 (Fig. 2d) and more macrophages were present in ADO2 lungs (Fig. 2e). The increased *Clcn7* expression in lungs was confirmed also by real time reverse transcription polymerase chain reaction (RT-PCR) (Supplemental Fig. 2b). Given the enrichment of the TFGβ signaling pathway (Fig. 1g), which is known to be involved in fibrosis, we tested the transcriptional expression of the TGFβ isoforms, the TGFβ-downstream genes αSMA and Grem2, and collagen I and III (Fig. 2f). We observed that, in ADO2 lungs, *TGFβ2*, *Grem2* and *Col1α1* chain were upregulated compared to WT lungs, suggesting a correlation between TGFβ pathway and perivascular fibrosis. The involvement of TGFβ was confirmed by immunofluorescence for its downstream transcription factor, pSmad2/3, which showed increased expression (Supplemental Fig. 3a) and nuclear translocation in ADO2 lungs (Fig. 2g). Similar results were observed in ADO2 kidneys (Fig. 2h–n, Supplemental Fig. 3b) and muscles (Fig. 2o–u, Supplemental Fig. 3c). However, in kidneys the number of ClC7-positive cells was very low compared to the total cell population, which could explain why the increase in pSmad2/3 expression was not detectable by Western blot (Supplemental Fig. 3b). Interestingly, expression of ClC7 (Fig. 2r) and macrophages (Fig. 2s) were observed in ADO2 but not in WT muscles, implying that the muscle was affected by the mutation in a myofiber non-autonomous manner. Although the TGFβ pathway is induced in ADO2 muscle, we were unable to demonstrate changes in pSmad2/3 compared to WT muscle (Fig. 2u, Supplemental Fig. 3c). These results suggest common pro-fibrotic alterations in ADO2 organs, albeit with some divergent features present in the downstream mechanism of the muscle.

ADO2 lungs also presented with lymphocytic infiltrations especially in the fibrotic perivascular regions (Supplemental Fig. 2a). Similar features were observed in kidneys (Supplemental Fig. 2c–e). ADO2 kidneys also showed elevated transcriptional expression of *Clcn7* (Supplemental Fig. 2f), along with an increased phosphoremia (Supplemental Fig. 2g), while calcemia (Supplemental Fig. 2h), uremia (Supplemental Fig. 2i) and uricemia (Supplemental Fig. 2j) were normal. Perivascular hypercellularity (Supplemental Fig. 2k) and increased transcriptional expression of *Clcn7* (Supplemental Fig. 2l) was observed in ADO2 muscles as well, although walking distance, measured by the open field (OF) test to assess muscular functionality, was unchanged (Supplemental Fig. 2m). Furthermore, ADO2 spleens showed more megakaryocytes than WT spleens (Supplemental Fig. 2n, o). Finally, *TGFβ1* and *Col1α1* were transcriptionally upregulated also in ADO2 compared to WT bone marrow (Supplemental Fig. 2p). Altogether, these results demonstrate that various visceral organs are affected by the *Clcn7*^*G213R*^ mutation, with a dominance of fibrosis especially in the perivascular tissues.

### Cellular phenotype

**Fig. 3:**
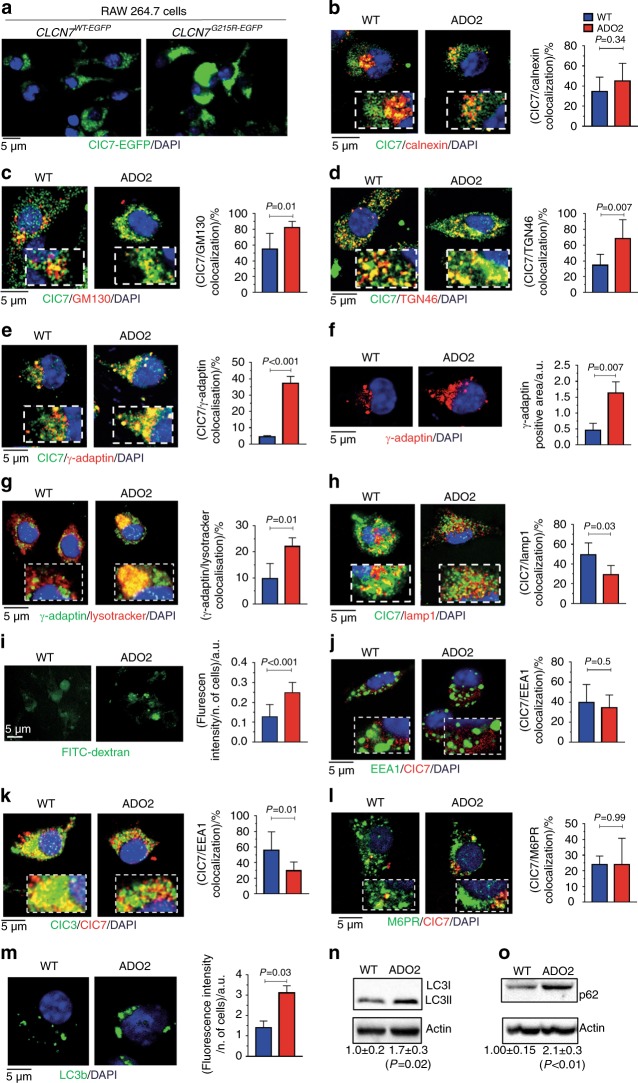
Cellular phenotype. **a** Fluorescence ClC7 expression pattern (green) in RAW 264.7 cells transfected with the indicated vectors. **b** Primary bone marrow mononuclear cells (BMMCs) were isolated from 10-day-old WT and ADO2 C57BL6/J. Immunofluorescence analysis of ClC7 (green) and the ER marker, calnexin (red) and quantification of ClC7/calnexin co-localization. **c** Immunofluorescence analysis of ClC7 (green) and the *cis*-Golgi marker, GM130 (red), and quantification of the ClC7/GM130 co-localization. **d** Immunofluorescence analysis of ClC7 (green) and the *trans-*Golgi marker, TGN46 (red), and quantification of the ClC7/TGN46 co-localization. **e** Immunofluorescence analysis of ClC7 (green) and the clathrin-coated vesicle marker, γ-adaptin (red), and quantification of the ClC7/γ-adaptin co-localization. **f** Immunofluorescence analysis of γ-adaptin (red), and quantification of γ-adaptin-positive area. **g** Immunofluorescence analysis of Lysotracker (red) and the clathrin-coated vesicle marker, γ-adaptin (green), and quantification of the Lysotracker/γ-adaptin co-localization. **h** Immunofluorescence analysis of ClC7 (green) and the lysosome marker, Lamp1 (red), and quantification of the ClC7/Lamp1 co-localization. **l** Analysis of the FITC–dextran fluorescence. **j** Immunofluorescence analysis of ClC7 (red) and the early endosome marker, EEA1 (green), and quantification of ClC7/EEA1 co-localization by ImageJ software. **k** Immunofluorescence analysis for ClC7 (red) and the late endosome marker, ClC3 (green), and quantification of the ClC7/ClC3 co-localization. **l** Immunofluorescence analysis for ClC7 (red) and the M6PR (green), and quantification of ClC7/M6PR co-localization. **m** Immunofluorescence analysis of the autophagosome marker, LC3b (green), and quantification of the LC3b fluorescence intensity. **n** Western blot analyses of the cell autophagy marker, LC3b (LC3I/LC3II) and **o** the cell autophagy substrate, p62/SQSTM (p62). Nuclei are stained in blue with DAPI. Images are representative, and graphs are the mean ± S.D of three experiments or five mice per group (Student’s *t* test)

To dissect the cellular mechanisms underlying the pathogenesis of organ alterations, we performed a series of studies by immunofluorescence and confocal microscopy. We transfected RAW 264.7 with the *CLCN7*^*G215R-EGFP*^ or the *CLCN7*^*WT-EGFP*^ vectors and observed by enhanced green fluorescent protein (GFP) fluorescence analysis an altered distribution of the mutant ClC7 compared to the WT protein. The mutant ClC7 was strongly accumulated in a paranuclear area, implying a defective protein trafficking (Fig. 3a). We, therefore, sought to investigate subcellular distribution and trafficking of ClC7 in WT and ADO2 primary bone marrow mononuclear cells (BMMCs), using a ClC7 antibody selected according to previous researches^16,23^ and validated in-house as described in Supplemental materials and methods.

The endoplasmic reticulum (ER) appeared normal in these ADO2 cells, with no changes in the co-localization of ClC7 with the ER marker, calnexin (Fig. 3b, Supplemental Fig. 5), and no difference in the expression of the ER stress proteins, GRp94, Bip1, and ERp57 (Supplemental Fig. 4a), compared to WT cells. In contrast, the co-localization of ClC7 with the *cis*-Golgi marker, GM130 (Fig. 3c, Supplemental Fig. 5), the *trans*-Golgi marker, TGN46 (Fig. 3d, Supplemental Fig. 5), and the clathrin-coated vesicle marker, γ-adaptin (Fig. 3e, Supplemental Fig. 5), as well as the paranuclear expression of γ-adaptin (Fig. 3f), were greater in ADO2 than in WT cells, suggesting an impaired vesicular transit through the Golgi stacks. In this context, we incubated the BMMCs with Lysotracker^®^, a fluorescent probe that stains the acidic vesicles in living cells. Cells were then fixed, gently permeabilized and immunostained for γ-adaptin. We observed that Lysotracker^®^ accumulated in the γ-adaptin-positive clathrin-coated vesicles of ADO2 cells, suggesting that this compartment exhibits a lower pH compared to WT cells (Fig. 3g, Supplemental Fig. 5). This result indirectly suggests that the mutant ClC7 keeps the chloride conductance capacity and that the pH in the γ-adaptin-positive clathrin-coated vesicles is likely to be decreased by the accumulation of the ClC7 shown in Fig. 3e. In contrast, the co-localization of ClC7 with the lysosome marker, Lamp 1, was reduced in ADO2 cells (Fig. 3h, Supplemental Fig. 5), leading to an increase of lysosome pH suggested by the stronger fluorescence of fluorescein isothiocyanate (FITC)-dextran, which is known to rise at high pH^24^ (Fig. 3i). Similar results were obtained using the Lysosensor^®^ probe, whose fluorescence switched from blue (lower pH) in WT cells to green (higher pH) in ADO2 cells (Supplemental Fig. 4b). Finally, we isolated lysosomes from WT and ADO2 cells and incubated them with neutral red, a probe that is up-taken by these organelles and retained inside their membrane upon protonation.^25^ The results showed that the neutral red content in ADO2 lysosomes was significantly lower than in WT lysosomes (Supplemental Fig. 4c), further suggesting an impairment of their acidification capacity.

While the co-localization of ClC7 with the early endosome marker, EEA1, was unchanged (Fig. 3j), the ClC7 co-localization with the late endosome marker, ClC3, was reduced in ADO2 BMMCs (Fig. 3k, Supplemental Fig. 5), whereas the co-localization of ClC7 with the Mannose-6P Receptor (M6PR) was normal (Fig. 3l, Supplemental Fig. 5), suggesting that ClC7 trafficking impairment did not involve its sorting receptor. Furthermore, the ClC7 β-subunit Ostm1 showed no difference in the co-localization with the Golgi marker TGN46 (Supplemental Fig. 4d), while its co-localization with the lysosome marker, Lamp1, was reduced (Supplemental Fig. 4e) in ADO2 vs. WT cells, suggesting impaired post-Golgi Ostm1 traffic as well.

**Fig. 4:**
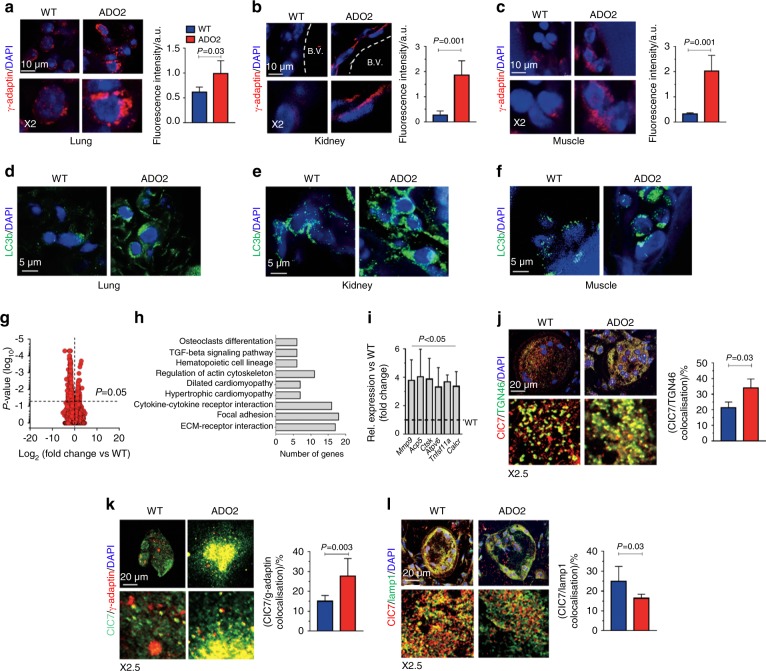
Cellular phenotype in in vivo lungs, kidneys, and muscles and in vitro osteoclasts. Lungs, kidneys, and muscles were harvested from 12-month-old WT and ADO2 CD1 male mice. **a** Immunofluorescence analysis of γ-adaptin (red), and quantification of γ-adaptin fluorescence in WT and ADO lungs **b** kidneys and **c** muscles. **d** Immunofluorescence analysis of LC3b (green) in lungs, **e** kidney and **f** muscles. **g** RNA-dSeq in osteoclasts differentiated from BMMCs of 10-day-old mice. Volcano plot of differential gene expression in ADO2 vs. WT osteoclasts. Horizontal dashed red line: *P* value threshold. **h** Pathway enrichment analysis using KEGG pathways database. **i** Relative expression of the indicated osteoclast differentiation markers extrapolated from RNA-dSeq data sets. **j** Immunofluorescence staining for ClC7 (red) and the *trans-*Golgi marker, TGN46 (green) in WT and ADO2 osteoclasts, and quantification of ClC7/TGN46 co-localization. **k** Immunofluorescence analysis for ClC7 (green) and γ-adaptin (red), and quantification of ClC7/γ-adaptin co-localization. **l** Immunofluorescence analysis for ClC7 (red) and the lysosome marker, lamp1 (green), and quantification of the ClC7/Lamp1 co-localization. Nuclei are stained in blue with DAPI. Images are representative, and graphs are the mean ± S.D of three independent experiments per group (Student’s *t* test)

Finally, ADO2 cells exhibited increased expression of the autophagosome marker, LC3b (Fig. 3m, n) and its partner protein p62 (Fig. 3o). Overall, these results demonstrated that the *Clcn7*^*G213R*^ mutation results in impairment of ClC7 Golgi exit, vesicular trafficking, lysosomal acidification and in altered autophagy in ADO2 cells. Interestingly, similar alterations were observed in ADO2 lungs, kidneys and muscles as exemplified by their increase of γ-adaptin (Fig. 4a–c) and LC3b (Fig. 4e, f) expression, suggesting shared pathogenic cellular mechanisms in vitro and in vivo.

Given that the osteoclasts remain the most recognized cell affected by ADO2 and that the cellular alterations induced by the *Clcn7*^*G213R*^ mutation have not been fully elucidated, we performed a RNA-dSeq of ADO2 and WT osteoclasts differentiated in vitro from the BMMCs by treatment with human recombinant (hr) macrophage-colony stimulating factor (M-CSF) and receptor activator of NF-κB ligand (RANKL), and observed several differentially expressed genes (Fig. 4g) belonging to various molecular pathways, including osteoclast differentiation and TGFβ signaling (Fig. 4h). Upregulation of osteoclast differentiation genes was also confirmed by real time RT-PCR (Fig. 4i) and could suggest that increased osteoclastogenesis is also an intrinsic ADO2 osteoclast feature, not only determined by the increase of parathyroid hormone known to contribute to the osteoclast-rich ADO2 phenotype.^26^ Furthermore, osteoclast-rich osteopetrosis is characterized by medullary fibrosis, which is severe in the homozygous *Clcn7* loss-of-function conditions.^16^ Therefore, along with the in vivo data showing transcriptional upregulation of the *Tgfβ* pathway and *Col1α1* in the ADO2 bone marrow (Supplemental Fig. 2p), the result in Fig. 4i could suggest that altered ADO2 osteoclasts could contribute to bone marrow fibrosis through the TGFβ signaling. Finally, ADO2 osteoclasts shared with BMMCs, lung, kidney, and muscle tissues impaired ClC7 Golgi transit (Fig. 4j), ClC7 accumulation into clathrin-coated vesicles (Fig. 4k) and reduced ClC7 expression in lysosomes (Fig. 4l). These results suggest that the cellular pathology is similar in the skeletal and the extra-skeletal ADO2 phenotypes induced by the *Clcn7*^*G213R*^ mutation.

### Neural phenotype

**Fig. 5:**
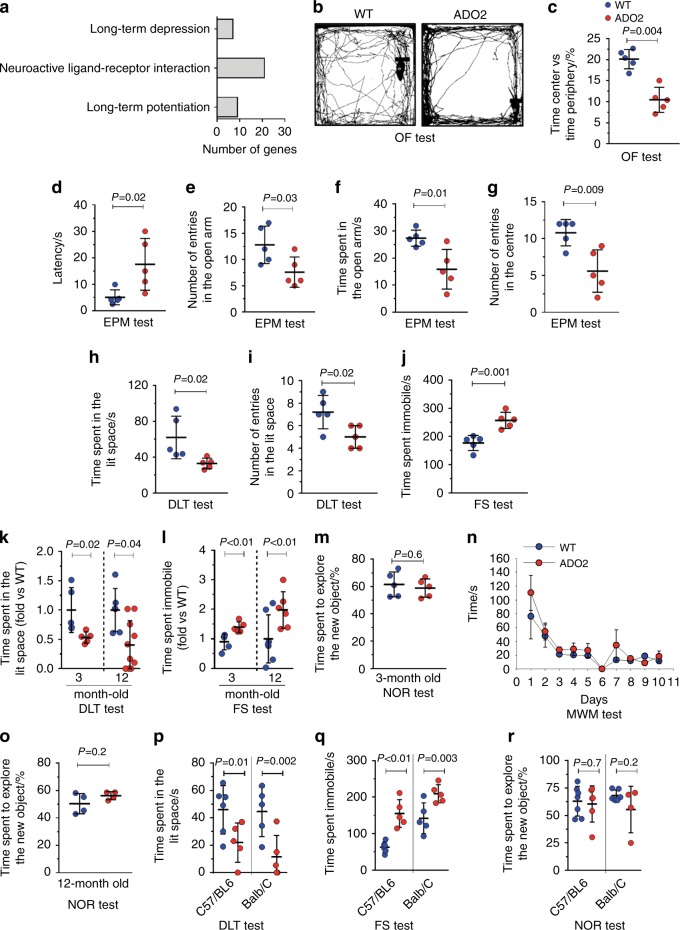
Behavioral and cognitive phenotype. **a** Enriched pathway analysis of RNA-dSeq datasets from 1-month-old WT and ADO2 C57BL6/J male mouse brains. Behavioral tests in 3-month-old WT and ADO2 CD1 male mice. **b** Representative tracks in the open field (OF) test. **c** Percentage of the time spent in the center vs. the time spent in the periphery of the OF arena. **d** Latency time, **e** number of entries in the open arm, **f** time spent in the open arm and **g** number of entries in the center measured by the elevated plus maze (EPM) test. **h** Time spent in the lit space and **i** number of entries in the lit space in the dark light transition (DLT) test. **j** Time spent immobile in the forced swimming (FS) test. **k** Three- and 12-month-old WT and ADO2 CD1 male mice compared for the time spent in the lit space during the DLT test and for **l** the time spent immobile during the FS test. **m** Novel object recognition (NOR) test and **n** Morris water maze (MWM) test in 3-month-old WT and ADO2 CD1 male mice. **o** NOR test in 12-month-old WT and ADO2 CD1 male mice. **p** DLT test in 12-month-old WT and ADO2 C57BL6/J and Balb/C male mice to measure the time spent in the lit space. **q** FS test in 12-month-old WT and ADO2 C57BL6/J and Balb/C male mice to measure the time spent immobile. **r** NOR test in 12-month-old WT and ADO2 C57BL6/J and Balb/C male mice to measure the time spent to explore the new object. Images are representative, and data are the mean ± S.D of five to eight mice per group (Student’s *t* test)

Handling of ADO2 mice proved challenging because of their jitteriness. Given the high expression of *Clcn7* in the brain (Fig. 1a) and the severe neurodegeneration observed in homozygous mutants,^16,27^ we hypothesized that a milder neural phenotype could be present in ADO2 mice as well. Consistent with our hypothesis, RNA-dSeq showed enrichment of pathways associated with long-term depression, neuroactive ligand–receptor interactions and long-term potentiation in ADO2 brains (Fig. 5a). Therefore, in order to test the anxiety-like behavior, mice were subjected to the OF, elevated plus maze (EPM) and dark and light transition (DLT) tests.^28,29^ The OF test measures not only the walking ability (see Supplemental Fig. 2m) but also the will to explore an open arena.^28^ We observed that while the distance traveled by WT and ADO2 mice was similar (Fig. 5b, Supplemental Fig. 2m), ADO2 mice spent less time in the center of the arena as opposed to the periphery when compared to WT mice (Fig. 5c), which is considered a sign of anxiety.^28^ In the EPM test,^28^ ADO2 mice showed a higher latency time to travel through the open arm of the apparatus than WT mice (Fig. 5d), while the number of entries in the open arm (Fig. 5e), the time spent in the open arm (Fig. 5f) and the number of entries in the center of the apparatus (Fig. 5g) were lower, further suggesting anxiety in ADO2 mice. Similarly, in the DLT test,^29^ ADO2 mice tended to spend less time in the lit compartment (Fig. 5h) and to limit the entrances in this compartment (Fig. 5i) compared to WT mice, again sign of anxiety. To test the depression-like phenotype, we performed the forced swim (FS) test,^28^ whose results revealed a greater time spent immobile by the ADO2 vs. the WT mice (Fig. 5j), thus suggesting a depressed behavior. By the DLT and the FS tests, we noted that anxiety and depression of ADO2 mice worsened with age (Fig. 5k, l). In contrast, cognitive tests, including the novel object recognition (NOR) test^30^ (Fig. 5m) and the Morris water maze (MWM) test^31^ (Fig. 5n), designed to investigate short-term and spatial memory, respectively, showed no differences between the genotypes. The NOR test also confirmed no changes between ADO2 and WT mice with ageing (Fig. 5o). Anxiety and depression shown in the CD1 mouse strain (Fig. 5b–l) were confirmed also in C57BL6/J and Balb/c strains by the DLT test (Fig. 5p) and the FS test (Fig. 5q), while the cognitive NOR test in these strains again showed no differences between the genotypes (Fig. 5r).

**Fig. 6:**
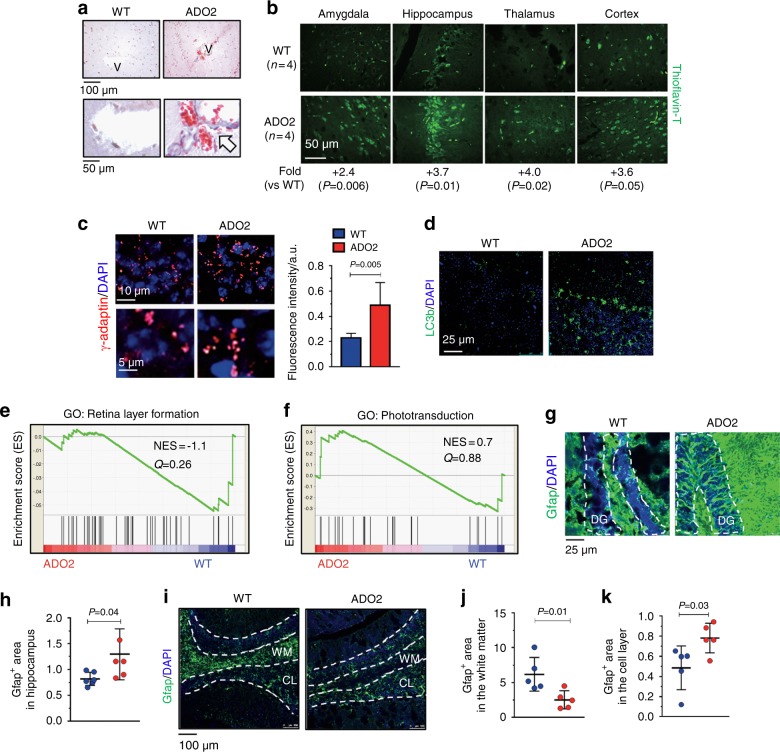
Neural phenotype. **a** Masson’s trichrome staining of brain sections of 12-month-old WT and ADO2 CD1 male mice. White arrow: perivascular collagen (blue) accumulation. V: vessels. **b** β-amyloid (green) detection in the indicated brain regions by Thioflavin-T fluorescence staining. **c** Immunofluorescence analysis of γ-adaptin (red) in the hippocampus of WT and ADO2 brain, and quantification (right panel). **n** Immunofluorescence analysis of LC3b (green) in the cerebellum. **e** Retina layer formation and **f** the phosphotransduction pathways in ADO2 vs. WT brains. **g** Immunofluorescence analysis of the astrocyte marker, Gfap (green) in hippocampus. Dashed white lines delimitate the dental gyrus (DG) of the hippocampus. **h** Quantification of the Gfap-positive area in the hippocampus. **i** Immunofluorescence analysis of Gfap (green) in the cerebellum, whose cortical areas are delimited by the dashed white lines. WM white matter. CL cellular layer. **j** Quantification of the Gfap positive area in the white matter and **k** in the cell layer of cerebellum by ImageJ software. Nuclei are stained in blue with DAPI. Images are representative, and graphs are the mean ± S.D. of five to eight mice per group (Student’s *t* test)

Histologically, we also noted perivascular fibrosis in ADO2 brains (Fig. 6a). Furthermore, investigating various areas of the brain by confocal microscopy, we noted an accumulation of β-amyloid (Fig. 6b) by staining with thioflavin-T, a benzothiazole dye that increases in fluorescence upon binding to amyloid fibers.^32,33^ Furthermore, increased expression of γ-adaptin (Fig. 6c) and LC3b (Fig. 6d) was noticed in ADO2 brains. Finally, RNA-dSeq of ADO2 vs. WT brains showed nonsignificant changes in retina layer formation genes (Fig. 6e) and phototransduction (Fig. 6f) pathways. These results suggest mild deterioration of the neural tissue in ADO2 mice with a cellular pathogenesis similar to that observed in vitro and in vivo in other cells and organs (see Figs. 3 and 4). ADO2 brains also exhibited astrogliosis, as demonstrated by increased expression of the glial fibrillary acidic protein (Gfap) both in the white and the grey matter of the hippocampus (Fig. 6g, h). Interestingly, Gfap expression was also higher in the cortex grey matter of the ADO2 cerebellum, but it was lower in the white matter compared to WT cerebellum (Fig. 6i–k).

### siRNA therapy for extra-skeletal alterations

**Fig. 7:**
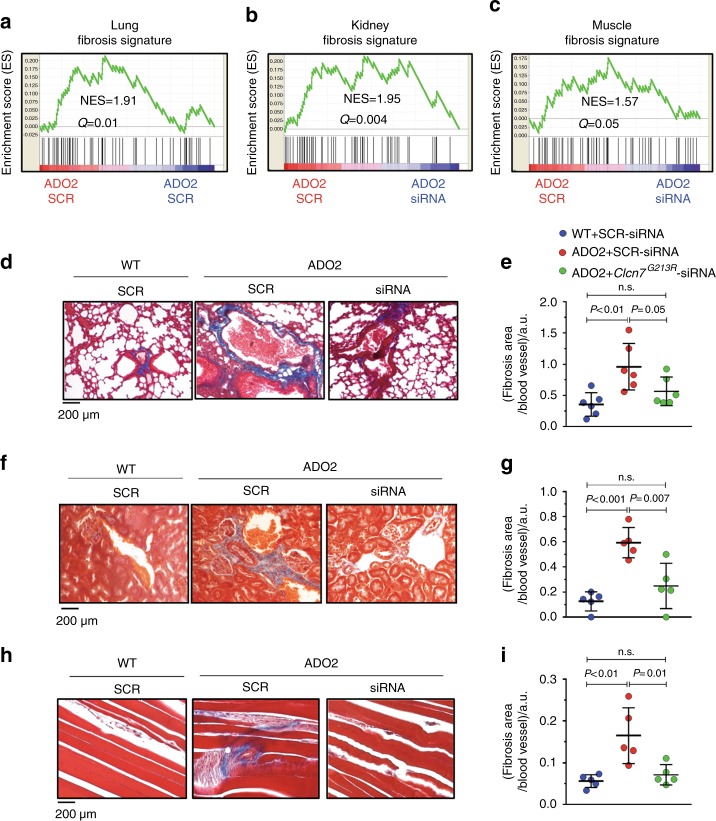
Effect of *Clcn7*^*G213R*^-siRNA treatment on fibrosis. **a** A 10-day-old WT and ADO2 C57BL6/J male mice were treated with 4 mg/kg of scrambled- (SCR) or *Clcn7*^*G213R*^-siRNA 3 times a week for 4 weeks.^19^ At the end of the experiments, mice were sacrificed and organs were harvested and used to extract RNA. Enrichment plots for fibrosis gene signature generated from ADO2 lung **b** kidney and **c** muscle RNA-dSeq data sets NES normalized enrichment score. **d** Three-month-old WT and ADO2 C57BL6/J male mice were treated as described in (**a**) for 12 weeks.^19^ At the end of the experiment mice were sacrificed and organs were harvested, fixed, and embedded in paraffin. Masson’ trichrome staining of lungs. **e** Quantification of the perivascular collagen (blue) accumulation. **f** Masson’ trichrome staining of kidneys. **g** Quantification of the perivascular collagen (blue) accumulation. **h** Masson’ trichrome staining of muscles. **i** Quantification of the perivascular collagen (blue) accumulation. Images are representative, and data are the mean ± SD of five mice per group (Student’s *t* test)

**Fig. 8:**
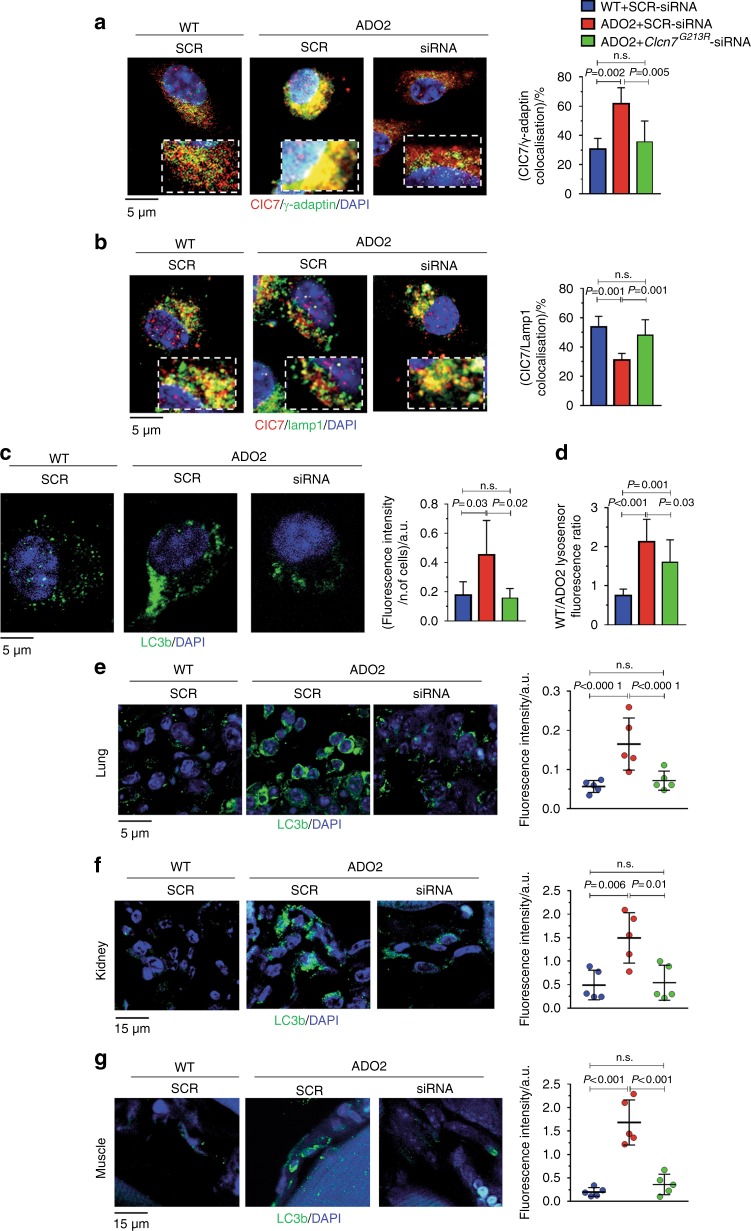
Effect of *Clcn7*^*G213R*^-siRNA treatment on the cellular defects. BMMCs isolated from 10-day old WT and ADO2 C57BL6/J mice and treated for 7 days with 100 nmol·L^−1^ of SCR- or *Clcn7*^*G213R*^-siRNA. **a** Immunofluorescence staining ClC7 (red) and γ-adaptin (green), and quantification of the ClC7/γ-adaptin co-localization. **b** Immunofluorescence analysis of ClC7 (green) and Lamp1 (red), and quantification of the ClC7/Lamp1 co-localization. **c** Immunofluorescence analysis of LC3b (green), and quantification of the LC3b fluorescence. **d** WT/ADO2 Lysosensor fluorescence ratio. **e** Immunofluorescence analysis of the cell autophagy marker, LC3b (green) in lungs from 3-month-old WT and ADO2 C57Bl6/J male mice treated with 4 mg·kg^−1^ of SCR- or *Clcn7*^*G213R*^-siRNA 3 times a week for 12 weeks, and quantification of LC3b fluorescence intensity. **f** Immunofluorescence analysis of LC3b (green) in the kidneys of mice treated as in (**e**), and LC3b quantification. **g** Immunofluorescence analysis of LC3b (green) in the muscles of mice treated as in (**e**), and LC3b quantification. Nuclei are stained in blue with DAPI. Images are representative, and graphs are the mean ± S.D. of five mice per group (Student’s *t* test)

We have demonstrated that a siRNA therapy restored the bone phenotype to normal in ADO2 mice.^17,19^ Using archive samples collected from our previous study, we evaluated the changes in extra-skeletal phenotypes in siRNA-treated ADO2 mice. Details of the study design have been reported in Maurizi et al.^19^ Briefly, 10-day-old and 3-month-old mice were treated with scrambled (control) and *Clcn7*^*G213R*^-specific siRNA (4 mg·kg^−1^, 3 times a week for 4 weeks by subcutaneous injection or 12 weeks by intraperitoneal injection, respectively), then extra-skeletal tissues were harvested and analyzed transcriptionally, histologically, and by confocal microscopy. Figure 7a–c shows an attenuation of the fibrosis signature in lung, kidney, and muscle tissues from *Clcn7*^*G213R*^-specific siRNA-treated compared to scrambled siRNA-treated ADO2 mice, as analyzed by RNA-dSeq. Furthermore, histological analysis confirmed the rescue of lung (Fig. 7d, e), kidney (Fig. 7f, g), and muscle (Fig. 7h, j) perivascular fibrosis in *Clcn7*^*G213R*^-specific siRNA-treated compared to scrambled siRNA-treated ADO2 mice. Moreover, BMMCs isolated from *Clcn7*^*G213R*^-specific siRNA-treated ADO2 mice exhibited normalization of expression of γ-adaptin (Fig. 8a), co-localization of ClC7 with Lamp1 (Fig. 8b) and expression of LC3b (Fig. 8c), as well as an improvement in vesicular acidification (Fig. 8d). Finally, immunofluorescence studies showed normalization of LC3b expression in lungs (Fig. 8e), kidneys (Fig. 8f), and muscles (Fig. 8g), suggesting efficacy of this innovative therapy also on the extra-skeletal alterations induced by the *Clcn7*^*G213R*^ mutation.

## Discussion

ADO2 has long been considered a benign form of osteopetrosis. Indeed, the disease is rarely lethal in adulthood, and most patients have a normal life expectancy.^4,5,12–14^ However, the term “benign” is now being revisited given the severe spectrum of symptoms that may affect patients. This misleading definition has very much limited the systematic medical evaluation of patients, who are generally diagnosed, monitored, and palliatively treated only for their bone alterations.^4^ Therefore, there is an unmet medical need that could represent a paradigm shift if it will be recognized that ADO2 is not simply a bone disease.

Our small cohort of ADO2 patients presented with fragmented clinical evaluations that showed variable, not well investigated and not well understood, extra-skeletal manifestations, and studies on larger cohorts were also inconsistent beyond the bone evaluation.^4^ We took advantage of our *Clcn7*^*G213R*^ knock-in mouse model^16^ to answer the question of whether *Clcn7*-dependent ADO2 exhibits exclusively a bone phenotype, which clashes with the notion of the wide tissue expression of *Clcn7*. Our results fit with this hypothesis and suggest that *Clcn7*-dependent ADO2 is a systemic condition affecting at least lung, kidney, brain, spleen, and bone marrow in an autonomous manner, and at least muscle through the recruitment of *Clcn7* expressing cells, apparently belonging to the macrophage family. Taken together, these results open a new perspective for forthcoming clinical and therapeutic developments.

Soft tissues in ADO2 appear to share a perivascular fibrosis. Macrophage and fibrotic signatures were observed in lungs, kidneys and muscles, and in all of them we also observed the induction of several pathways associated with the onset of tissue fibrosis, including the PI3K/Akt, PPAR, RAS, cAMP, and TGFβ signaling, in organs such as lungs and kidneys.^34–37^ Enrichment of RAS and cAMP signaling pathways was also found in brain, in line with the perivascular fibrosis observed in this tissue as well, suggesting a partially overlapping mechanism shared by the brain and the other organs.

In lungs, kidneys, and muscles, our research focused on the TGFβ pathway, because it is a potent inducer of fibrosis also involved in the impairment of autophagy.^38–41^ Macrophages^42^ and TGFβ signaling^41,43^ converge on tissue fibrosis. Macrophages belong to the same family as osteoclasts and it is not surprising that they express high levels of ClC7 given their phagocytic activity requiring extensive lysosome involvement.^44^ They share many molecular pathways with osteoclasts, including expression of the c-Fms receptors for M-CSF,^45^ expression of immune-receptor-tyrosine-based-activation motif co-receptors^46^ and very intense acidic hydrolase vesicular trafficking to the lysosomes.^47^ Our results suggest that the macrophages can play a leading role in the multi-organ dysfunction in *Clcn7*-dependent ADO2, as demonstrated by the perivascular fibrosis observed in ADO2 muscle, which express very low autogenous *Clcn7* but presents with increased numbers of *Clcn7*-positive macrophages, especially in the perivascular areas. Given the recognized role of macrophages in tissue fibrosis,^42^ we believe that this event is essential for the observed muscle tissue alteration in ADO2.

With the exception of muscle, all other organs investigated in this study expressed high levels of *Clcn7*. Nevertheless, in all organs we also observed perivascular fibrosis and increased cellularity, which included lymphocyte infiltrates and macrophages. Therefore, it is conceivable to hypothesize that in lung, kidney, spleen, and bone marrow, macrophages could contribute to worsen the autogenous alterations induced by the resident cells. For example, homozygous *Clcn7*^*G213R*^ mice die within 1 month of birth and this has been considered to be solely the effect of severe marrow failure and neurodegeneration induced by the *Clcn7* loss-of-function mutations. However, our observations that lungs express high levels of *Clcn7* in the bronchiolar epithelium and in the alveolar macrophages, and that homozygous mutant lungs present with severe alveolar atelectasis and air way closure, suggest a lung-autonomous failure induced by the *Clcn7* mutation in resident cells. ADO2 is not considered a disease affecting the brain, as opposed to *CLCN7*-depended autosomal recessive osteopetrosis in which severe neurodegeneration and lysosomal storage disease can be observed in patients.^1,11,48^ However, a few ADO2 patients were reported to present with cognitive failures.^4,14^ Systematic studies on brain involvement in ADO2 patients are not available and manifestations like anxiety and depression, if noted, may be underestimated as they could be nonspecifically associated with any illness status. Our ADO2 mice offered us the opportunity to investigate the brain phenotype in-depth. We noted no obvious histological signs of neurodegeneration,^16^ as opposed to the homozygous *Clcn7* knock-out^21,27,49^ and *Clcn7*^*G213R*^ homozygous knock-in^16^ mice that recapitulate the clinical manifestations of autosomal recessive osteopetrosis, but we observed behavioral changes associated with anxiety and depression. In agreement with these observations, RNA deep-sequencing in brain revealed modulation of genes associated with long-term depression, long-term potentiation and neuroactive ligand–receptor interaction signaling pathways. Contrary to the knowledge in humans, we did not observe, in our experimental conditions, evidence of cognitive failure in ADO2 mice. However, since cognitive failures in humans are very rare,^4,14^ speculatively they may be associated only with extreme phenotypes. Unfortunately, no epidemiological studies are available on human ADO2 that could support or deny our observation. Nevertheless, we confirmed a brain phenotype in ADO2 mice, exhibiting perivascular fibrosis, accumulation of β-amyloid and astrogliosis.

The observations in our study favor the hypothesis of an organ autonomous onset in response to the mutant ClC7. This concept is supported by the high expression of *Clcn7* in all organs investigated but the muscle. However, even in muscle, recruitment of highly expressing ClC7 macrophages supports a local response also in this tissue. Our global *Clcn7*^*G213R*^ knock-in mouse model does not allow to rule out that organ responses might also be induced by the bone alterations, although they were observed also in the CD1 mouse strain that showed very moderate bone manifestations.^16^ Conditional tissue-specific *Clcn7*^*G213R*^ knock-in strategy would help addressing this important question, but this model is not available yet.

The cellular alterations induced by the *Clcn7*^*G213R*^ mutation are still unclear. Transfecting the GFP-tagged human *CLCN7*^*G215R*^ homolog in CHO cells, Schulz et al.^50^ found high-ER localization of the protein and normal chloride conductance. In contrast, Henriksen et al.^51^ showed unaltered ClC7 subcellular localization but impaired chloride conductance in human ADO2 osteoclasts generated by peripheral blood mononuclear cells. Moreover, Kajiya et al.^52^ showed that the G215R mutation reduces the acid-activated chloride conductance of the ClC7 channel in HEK293 cells transfected with the GFP-tagged human *CLCN7*^*G215R*^ compared to the WT construct. Our cellular studies showed instead that ClC7 accumulates in the Golgi, both in vitro and in vivo, in all ADO2 cell types tested, strongly suggesting that the ClC7 Golgi exit pathway is impaired, dampening the downstream trafficking to the target organelles. As a consequence of the accumulation in the Golgi, the ClC7 protein could not transit to the lysosomes and the lysosomes could not properly acidify their content. It has to be noted that Kasper et al.^27^ and others^53–57^ showed a normal pH and/or lysosomal acidification in ClC7/OSTM1-deficient lysosomes. This observation is not in contrast with our study given that in their model the protein was deleted and speculatively the acidification could be compensated by other mechanisms transiting to the lysosomes. Moreover, in line with the previous findings that the ClC7 β-subunit OSTM1^58^ requires the ClC7 to be exported from the ER,^53,59^ the lysosomal localization of the OSTM1 was reduced in ADO2 cells. In contrast, OSTM1 localization in the *trans*-Golgi was unchanged, suggesting that the accumulation of ClC7 in the Golgi also impaired the post-Golgi trafficking of OSTM1. Interestingly, the clathrin-coated vesicles enriched in mutant ClC7 were able to acidify their content, suggesting that its ion transport function was retained. Therefore, the major alteration of the mutant ClC7 could be associated with the mislocalization of the protein rather than with its functional impairment.

Lysosome homeostasis is indispensable for controlling autophagy,^60^ therefore it is not surprising that the expression of the autophagosome marker, LC3b, and its partner protein, p62, were deregulated in ADO2 cells. These findings are in line with the observation by Wartosch et al.,^21^ who found an increased LC3II level in the brain and in the kidney of the *Clcn7* knock-out mice. Given that p62 is a multidomain protein also implicated in the activation of the transcription factor NF-κB,^61^ and that NF-κB is downstream of the most potent osteoclastogenic cytokine, RANKL,^62,63^ we can speculate that the increased osteoclastogenesis in *CLCN7*-dependent ADO2 could also be mediated by the high expression of p62. It has to be noted that the co-localization analysis by confocal microscopy has limitations due to the intrinsic differences in the fluorescence intensity detected in each cellular preparation. However, differences between ADO2 and WT cells were consistent and statistically significant over the experiments, representing an asset of the study.

Given that ADO2 occurs due to a missense mutation of a dimeric protein,^64^ in our previous work we demonstrated in mice that it is curable by a specific siRNA that silences the mutant gene without affecting the expression of the normal allele.^17,19^ This patent-protected therapy (Patent application PCT/IB2015/053730), proved to be safe and effective in rescuing the ADO2 bone phenotype employing various treatment regimens in young, adult and aging mice, in males and females and by different routes of administration.^17,19^ In this study, we demonstrated that the therapy has systemic efficacy rescuing the perivascular fibrosis observed in lungs, kidneys, and muscles, while also restoring the normal vesicular trafficking, the lysosomal acidification ability and the autophagy pattern in vitro and in vivo. This result further strengthens the innovation of our experimental therapy that can be envisaged to be suitable to cure not only the skeletal but also the extra-skeletal *CLCN7*-dependent ADO2 manifestations.

In conclusion, we demonstrated that *Clcn7*-depedent ADO2 not only is a bone disease but also affects other organs with similar pathogenesis and cellular alterations. Furthermore, these extra-skeletal manifestations are experimentally cured by a specific siRNA therapy already proven to rescue the bone phenotype of ADO2 mice. We believe that these findings have important clinical implications that in the future might translate into benefits for patients in terms of correct diagnosis, effective follow up and new targeted therapeutic options, one of which could be represented by our experimental siRNA therapy. Lastly, we propose that systematic epidemiological studies are necessary to confirm the concept of multi-organ involvement in the pathogenesis of human *CLCN7*-dependent ADO2.

## Material and methods

### Animals

All in vivo experiments were conducted in agreement with the national and international guidelines and policies (European Economic Community Council Directive 86/609, OJ L 358, 1, December 12, 1987; Italian Legislative Decree 4.03.2014, n.26, *Gazzetta Ufficiale della Repubblica Italiana* no. 61, March 4, 2014) and were approved by the Italian Ministry of Health (n° 564/2016-PR). The study was performed according to the Animal Research: Reporting of In Vivo Experiments (ARRIVE) guidelines, upon randomization of the mice, with usually ≥5 mice/group, unless otherwise stated. Mice were humanely sacrificed by CO_2_ inhalation.

The *Clcn7*^*G213R/WT*^ (ADO2) mouse model (*Mus Musculus*, C57BL6/J, CD1 and Balb/C backgrounds) has been described in Alam et al.^16^ This ADO2 animal model carries the mouse homolog of the most frequent human ADO2 mutation, *CLCN7*^*G215R*^, recapitulating the human ADO2 phenotype.

### RNA-dSeq and bioinformatics analyses

RNA was isolated from WT and ADO2 lungs, kidneys, muscles, spleens, bone marrows, and brains, and from 10^6^ WT and ADO2 osteoclasts differentiated in culture, using the RNAeasy mini kit. After isolation the RNA concentration and the A_260/280_ ratio were checked by Nanodrop. RNA quality was checked by 1% agarose gel run and with Bioanalyzer system (Agilent). The RNAs were then precipitated in ethanol and sent to the GATC Biotech (Germany) for the analysis. RNA was isolated from tissues using the RNAeasy mini kit starting from 10 mg of tissue. After isolation the RNA concentration and the A_260/280_ ratio were checked by Nanodrop. RNA quality was checked by 1% agarose gel run and with Bioanalyzer system. The RNAs from organs of 5 mice/group were pooled together and the RNA pools were then precipitated in ethanol and sent to GATC Biotech for the RNA-dSeq analysis.

RNA-dSeq reads were aligned to the reference transcriptome (mm10/GRCm38, Ensembl; v85 Ensembl) using Bowtie transcriptome alignments. TopHat identified the potential exon–exon splice junctions of the initial alignment. Then Cuffinks identified and quantified the transcripts from the preprocessed RNA-dSeq alignment assembly. After this, Cuffmerge merged the identified transcript pieces to full length transcripts and annotates the transcripts based on the given annotations. Finally, merged transcripts from two or more samples were compared using Cuffdiff to determine the differential expression levels at the transcript level between samples.

After the analyses, the generated RNA-dSeq data sets, containing the expression profile of 36 000 genes for each sample/condition, were used for the enrichment analyses performed using the free online tool KEGG Pathway or the GSEA software. The GSEA enrichment analysis generated an enrichment score (ES), which reflected the degree to which a gene set was overrepresented at the top or bottom of a ranked list of genes. The normalized enrichment score was generated by the formula below, normalizing the ES for the differences in gene set size, taking into account all possible permutations of the dataset.

$${\mathrm{NES = }}\frac{{{\mathrm{ES}}}}{{{\mathrm{Means}}\,\left( {{\mathrm{ES}}\,{\mathrm{against}}\,{\mathrm{all}}\,{\mathrm{permutation}}\,{\mathrm{of}}\,{\mathrm{the}}\,{\mathrm{data}}\,{\mathrm{set}}} \right)}}{.}$$

The statistical methods used for the analyses are described in the statistical analysis paragraph of the materials and methods section and stated in the figure legends. The GSEA gene sets databases are described in the Supplemental materials (Supplemental Table 1).

### Immunofluorescence in primary BMMCs and osteoclasts

Primary BMMCs or osteoclasts were fixed in 4% paraformaldehyde for 8 min, permeabilized with PBS-Triton X-100 0.05% and blocked with 3% bovine serum albumin–PBS for 30 min at room temperature. Cells were then incubated with a single or multiple primary antibody mixture for 1 h at room temperature and then overnight at 4 °C. Secondary incubations were for 1 h at room temperature with the corresponding secondary antibodies at dilution 1:1 000. Sections were mounted with DAPI antifade mounting medium. Immunofluorescence quantification and co-localization analyses were done using Fiji^®^ software. The script used for this analysis is reported in Supplemental Table 4. The raw data of the co-localization analysis are reported in Supplemental Table 5.

### Measurement of intracellular acidity

BMMCs (WT and ADO2) were seeded in 24 well-plates (cell density: 70 000/well) at least 18 h before the time of imaging. Cells were incubated with DMEM containing 1 μmol·L^−1^ LysoSensor Yellow/Blue DND-160 probe for 5 min at 37 °C and 5% CO_2_. After incubation, BMMCs were kept in HBBS supplemented with 25 mmol·L^−1^ HEPES. To evaluate the intracellular acidity, cells were observed in blue (W1; 417 nm–483 nm) and yellow (or green) (W2; 490 nm–530 nm) and 100× images were collected over a period of 5 min for each sample, using the epifluorescence microscope Zeiss AxioPlan and the Axiovision Software^®^. Then, Lysosensor fluorescence ratio (W1/W2) images were generated according to Kardash et al.,^65^ and the fluorescence ratio values were calculated for the test samples by the calculator function of the Fiji^®^ software (Supplemental Table 4).

Cells were also loaded with 0.5 mg·mL^−1^ of FITC–Dextran (MW 10 000) in RPMI medium supplemented with 10% fetal bovine serum (FBS) for 2 h at 37 °C and 5% CO_2_ (pulse phase). After 2 h, medium was replaced with RPMI supplemented with 10% FBS and cells incubated for further 2 h (chase phase)^54^ and then washed with PBS. 40× images were acquired. Also, the analysis of the FITC fluorescence intensity was done using the Fiji^®^ software (Supplemental Table 4).

### Behavioral and cognitive tests

Behavioral and cognitive tests were performed by standard procedures. Behavioral analysis, included OF,^28^ EPM,^28^ DLT,^29^ and FS^28^ tests. Cognitive analysis, included NOR^30^ and MWM^31^ tests. Details are described in Supplemental materials and methods.

### siRNAs preparation and in vivo treatments

The sequence of the custom-made siRNA against the *Clcn7* ^*G213R*^ used for the in vivo treatment, is published in Capulli et al.^17^ and protected by the patent application PCT/IB2015/053730. The siRNA had modified 3′dAdT overhangs to enhance the conjugation with the in vivo-jetPEI^®^ transfection reagent used as vehicle. The formulation was done as suggested by the manufacturer. Mice were treated with 4 mg·kg^−1^ of control scrambled siRNA (SCR) or *Clcn7* ^*G213R*^-siRNA (siRNA) intraperitoneally or subcutaneously, 3 times a week for 4 or 12 weeks, as stated in the figure legends. All treatments were done in male mice.

### Statistical analysis

All results are presented as mean ± SD of at least five mice/group, unless otherwise indicated in the figure or table legends. Only data obtained by real time RT-PCR were normalized and shown as fold changes to control for unwanted sources of variation. Statistical analyses were carried out using the unpaired, two-tailed Student’s *t* test, or Multiple Comparison ANalysis Of VAriance (Supplemental Table 3) with the software Prism^®^ by GraphPad v7.0. *P* values threshold was < 0.05.

Data from RNA-dSeq analyses on tissues are representative of one mRNA data set derived from a pull of mRNAs isolated from five mice per group. Data from RNA-dSeq analysis of osteoclasts are representative of three mRNA data sets derived from three independent osteoclasts cultures. For statistics, an uncorrected *P* value generated by the Cuffdiff analysis was used. The statistical significance for the enrichment analyses was computed using an FDR-adjusted *P* value (*Q* value). A *Q* value ≤ 0.1 has been considered statistically significant. The statistical methods used for the analyses are reported in the figure legends and in Supplemental Table 3.

## Supplementary information

Supplemental material
