# Supplementary material for: Extra-skeletal manifestations in mice affected by Clcn7-dependent autosomal dominant osteopetrosis type 2 clinical and therapeutic implications
Source: Bone Res. 2019 Jun 11;7:17. doi: 10.1038/s41413-019-0055-x (PMC6559989; doi:10.1038/s41413-019-0055-x)
Supplement: Supplementary file 1 — Supplemental material [file 41413_2019_55_MOESM1_ESM.pdf]

Supplemental Materials and methods

Scrambled siRNA and siRNAs specific for the *Clcn7*^{G213R} mutant (Capulli *et al.*[1]; Patent application PCT/IB2015/053730, publication number WO2015177743 A1) were custom-made from GE Dharmacon (Lafayette, CO, USA) along with three ON-TARGETplus SMART pool siRNAs specific for *Clcn7* (cat# L-059174), *Clcn3* (cat# L-044441) and *Ostm1* (cat# L-057484. . Plastic ware was from Falcon Becton-Dickinson (Cowley, Oxford, UK). Dulbecco's modified Minimum Essential Medium (DMEM) (cat# ECB7501L), Hank's Balanced Salt solution (HBBS) (cat# ECB4007L) and Phosphate Buffered Saline (PBS) (cat# ECB4004L) for cell culture were provide by EUROCLONE (Milan, Italy). Fetal Bovine Serum (FBS) (cat#26140-079), Lipofectamine (cat# 11668027), Trizol® reagent (cat# 15596026) and primers were from Invitrogen, Carlsbad, CA). Human recombinant (hr) Receptor Activator of Nuclear Factor κ-light-chain-enhancer of activated B cells transcription factor Ligand (RANKL) (cat#310-01) and hr Macrophage-Colony Stimulating Factor (M-CSF) (cat#300-25) were from Peprotech (EC, London). RevertAid H Minus First Strand cDNA Synthesis Kit (cat# K1631) and LysoSensor™ Yellow/Blue DND-160 (cat# L7545) were from Thermo Scientific (Waltham, Massachusetts, USA). The SensiMix™ SYBR® kit was from Bioline (cat# QT605, London, UK). Masson's trichrome kit was from Bio Optica (cat# 04-010802). RNAeasy mini was provide by Qiagen (cat# 74104, Hilden, Germany). DAB+ substrate Chromogen System (DAKO, cat# k0679). Calcium (cat#ab102505) and phosphate (cat# ab65622) colorimetric assays were provide by Abcam (Cambridge, UK). VECTASTAIN Elite ABC HRP Kit was from Vector Laboratories (Burlingame, California, USA). The transfection reagent *in vivo*-jetPEI® was from Polyplus-Transfection (cat# 201-50G, Illkirch, France). The Reflotron® kits were from Roche Diagnostics (Manheim, Germany). Unless indicated otherwise, all the other reagents were of the purest grade from Sigma Aldrich Co. (St. Louis, MO, USA).

Real time RT-PCR

Total RNA was extracted from mouse tissues or cells using Trizol® reagent and according to the protocol suggested by the manufacturer. After quantification by Nanodrop (Thermo Scientific), the RNA quality was checked by 1% agarose gel run. For cDNA synthesis 1 µg of RNA was then reverse transcribed using the

RevertAid H Minus First Strand cDNA Synthesis Kit. Real time PCR reaction was performed loading 0.1µg of cDNA using the SensiMix™ SYBR® kit. Gene expression data were represented as fold change over the control and normalized by *gapdh*, unless otherwise stated. Primers sequences are listed in Supplemental information (Supplemental Table 6).

Histology and histomorphometry

Soft tissues were fixed in 4% paraformaldehyde for at least 48 hours and processed for paraffin or Optimal Cutting Temperature (OCT) embedding. Microtome or cryotome sectioning were used to obtain tissue slices of 5 and 10 µm thickness, respectively. Hematoxylin and Eosin and Masson's trichrome staining were used to evaluate tissue morphology and fibrosis. Histomorphometry was performed using Fiji® software to evaluate tissue fibrosis and cellular infiltrates.

Immunostaining in mouse tissues

For immunohistochemistry, sections were deparaffinized, endogenous peroxidases were quenched with 3% H_2O_2 and the non-specific binding sites blocked with 5% BSA. Sections were incubated for 1 hour at room temperature and then overnight at 4°C with anti-ClC7 goat or rabbit polyclonal antibody (Santa Cruz Biotechnology). Vectastain Elite ABC HRP Kit was used to develop the immunostaining following the manufacture's instruction and signal was detected by DAB. Section were counterstained with hematoxylin, then dehydrated and mounted.

For immunofluorescence on paraffin-embedded samples, sections were deparaffinized, permeabilized with PBS with Tween 20 (PBS-T) 0.1%, blocked with 5% Bovine Serum Albumin (BSA) and incubated with a single or multiple primary antibodies mixture 1 hour at room temperature and then overnight at 4°C. Secondary incubations were for 1 hour at room temperature with corresponding secondary antibody at dilution 1:1,000. Sections were mounted with 4',6-Diamidine-2'-phenylindole dihydrochloride (DAPI) antifade mounting medium.

For immunofluorescence on OCT-embedded samples, cryosections were rehydrated in PBS for 30 minutes and incubated for 2 hours with blocking/permeabilization solution (5% BSA in 0.1% PBS-Triton X-100) at room temperature. Sections were then incubated with a single or multiple primary antibodies mixture for 1 hour at room temperature and then overnight at 4°C. Secondary incubations were for 1 hour at room temperature with corresponding secondary antibody at dilution 1:1,000. Sections were mounted with DAPI antifade mounting medium.

The list of the antibodies and the dilutions used are reported in the Supplemental materials (Supplemental Table 3).

Serological tests

Biomarkers of kidney disease were measured in the sera of 12-month-old CD1, using specific colorimetric assays for calcium and phosphate, and diagnostic Reflotron® strips for urea and uric acid, the following the manufacturer's instructions[2].

Primary bone marrow mononuclear cell (BMMCs) and osteoclast cultures

For BMMCs isolation, bone marrow was flushed out from the bone cavity of the long bones of 10-day-old mice (C57BL6/J background) and diluted 1:1 in HBBS, layered over Histopaque 1077 solution and centrifuged at 400*g* for 30 minutes. Cells were washed twice with HBBS, re-suspended in DMEM + 10% FBS and plated in culture dishes. After 12 minutes, the non-adherent cells were harvested and counted. BMMCs were plated in 6 or 24 well-plate dishes, with or without round glass coverslips, to perform protein or RNA isolation and immunofluorescence staining, respectively. Cells were maintained for 7 days in DMEM + 10% FBS, supplemented with 50 ng/ml rhM-CSF.

For osteoclast generation, BMMCs obtained as described in the previous paragraph were plated in 10 cm or

24 well-plate dishes, with or without round glass coverslips, to perform RNA isolation and immunofluorescence staining, respectively. The cells were maintained for 7 days in DMEM + 10% FBS supplemented with 50 ng/ml human recombinant (rh)M-CSF and 120 ng/ml rhRANKL.

Neutral red uptake assay

Lysosomes were isolated from bone marrow flushed out from long bones of 2-month-old WT and ADO2 mice using the lysosome isolation kit (LYSISO1) provided by Sigma Aldrich, according to the manufacturer's instructions. After isolation, lysosomes were exposed to Neutral Red dye (Catalog Number N2537) and the uptake was followed by a spectrophotometer in real time for 10 minutes. The maximum absorbance of the Neutral Red dye shifts from 460 nm to 510 nm in acidic pH conditions. The Neutral Red uptake was calculated using the following formula:

$$\text{Neutral Red uptake} = \Delta A/\text{min}_{(510)} - \Delta A/\text{min}_{(460)}$$

Protein extraction and Western blot

BMMCs or tissues were lysed in standard RadioImmunoPrecipitation Assay (RIPA) buffer (1M Tris/HCl, pH 7.4, 1M NaCl, Nonidet P-40, 10% sodium deoxycholate, 0.5M EDTA, pH 8, 0.1M NaF, 20mM Na_3VO_4, dH_2O, 0.1M PMSF) containing 1% protease inhibitor cocktail and 10 µM sodium fluoride. Proteins concentration was quantified using the Bradford assay. Protein lysates (20-30µg) were resolved by SDS-PAGE, immunoblotted with a primary antibody reported in Supplemental Table 3, overnight at 4°C, and detected by HorseRadish Peroxidase-(HRP)-conjugated secondary antibodies (all at 1:2,000 dilution) and enhanced chemiluminescence on a ChemiDoc® imaging system. Densitometric analyses were performed using Image Lab® software provided by Bio-Rad.

Behavioral and cognitive tests

For the behavioral tests, male mice of both genotypes (WT and ADO2) were kept in the testing room the day before the tests, at standard laboratory condition. All the tests were done between the 10 a.m. and 4 p.m.

and mice were transported in their home cages. The apparatus used for the tests were cleaned with 50% ethanol before and at the end of each session or test. The tests were conducted blindly, and the mouse genotype was checked at the end of the test.

Open Field (OF) test[3]. This test was used to assess anxiety-like behavior and locomotor activity. Mice were put in the center of a 50x50 cm plastic box and their activity was recorded for 30 minutes using a video system. After the test, the videos were analyzed using the video tracking software Kinovea (version 0.8.15) to measure the total walking distance and the time spent in the center and in the periphery of the OF arena by the mice.

Elevated Plus Maze (EPM) test[3]. This test was used to assess anxiety-like behavior. The EPM apparatus was made of plastic and consisted of four arms, two enclosed and two open, each measuring 30 cm in length and elevated 50 cm from the floor. Each mouse was placed in the center of the maze and allowed to explore the apparatus for 5 minutes. During the test the number of entries and the time spent in the open arms were measured by Stopwatch+ software (Version 1.5.1). The time spent in the center of the apparatus and the latency were recorded as well.

Dark and light Transition (DLT) test[4]. This test was used to assess anxiety-like behavior. It was performed using a 30x60 cm plastic box divided in two compartments: the lit and the dark compartments. Mice were put individually into the dark compartment facing away from the doorway to the light compartment. The number of entries and the time spent in the lit compartment were recorded for 5 minutes using Stopwatch+ software (Version 1.5.1).

Forced Swimming (FS) test[3]. This test was used to assess depression. During this test mice were placed individually into glass cylinders containing 10 cm deep water warmed to 25°C. Animals were tested for 6 minutes and the time spent immobile recorded with Stopwatch+ software (Version 1.5.1). Mice were

considered immobile when they made no attempts to escape, except for the movements necessary to keep their heads above the water.

Novel Object Recognition (NOR) test[5]. This test was used to assess the short-term memory. A plastic box was divided into two spaces with same dimension (30x20 cm each). This configuration of the apparatus allows two mice to run simultaneously avoiding the contact during the test. To test the short-term memory of the mice, a black plastic object (object 1) and a clear plastic funnel (object 2) were used. The test was divided into 3 sessions run on three different days. During day 1, mice were placed into the box without the objects for 5 minutes; on day 2 mice were exposed to two similar objects (object 1) for 10 minutes to allow them to familiarize with the object. Finally, on day 3, one of the old objects (object 1) was replaced with the new one (object 2), letting the mice to explore the novel object for 15 minutes. All the sessions were recorded, and the time spent to explore the novel and the old objects was calculated using the Stopwatch+ software (Version 1.5.1).

Morris Water Maze (MWM) test[6]. This test was used to assess spatial memory. The maze had a diameter of 150 cm and contained warmed water (22-23°C) made opaque with non-toxic white paint. The pool was equipped with distal visual cues, including geometric figures attached to the walls, which were used by the mice for the spatial orientation. Mice were assessed across repeated trials (4 trials/day for 10 days). During these trials, a 15 cm round platform was hidden 1 cm beneath the surface of the water at a fixed position. Each daily trial consisted of four swimming trials starting randomly from each of four starting positions. The duration of each trial was 2 minutes and mice that failed to find the platform within this time were guided to the platform. Mice had to remain on the platform for 15 seconds before they were returned to their cages. The MWM test were done on 10 subsequent days. The time spent by the mice to find the hidden platform was recorded using Stopwatch+ software (Version 1.5.1).

Validation of antibodies

The antibodies against ClC7, ClC3 and OSTM1, for which there was poor information on specificity, were validated in this study as follows: ClC7 by Western blot in WT and ADO2 cells (Supplemental Figure 4f) and by immunofluorescence in scrambled-siRNA and *Clcn7*-siRNA treated primary WT BBMCs (Supplemental Figure 4g); ClC3 and OSTM1 antibodies were validated by immunofluorescence in scrambled-siRNA or *Clcn3*-siRNA and *Ostm1*-siRNA treated primary WT BBMCs (Supplemental Figure 4g).

Supplementary tables

Supplementary table 1 – GSEA data sets

Dataset	Genes in the dataset
Macrophage Signature	1100001G20Rik;1110007C09Rik;1110018G07Rik;1110046J04Rik;1190003J15Rik;1200009I06Rik;1300002K09Rik;1600010M07Rik;1600029D21Rik;1700112E06Rik;1810011H11Rik;1810029B16Rik;1810033B17Rik;2010002N04Rik;2010109K09Rik;2010109K11Rik;2210011C24Rik;2210406H18Rik;2310008H09Rik;2310014L17Rik;2310016C08Rik;2310028H24Rik;2310044G17Rik;2400009B08Rik;2500002B13Rik;2610002D18Rik;2610021K21Rik;2810417H13Rik;2900076A13Rik;3110027N22Rik;3110043O21Rik;4632428N05Rik;4632434I11Rik;4833419A21Rik;4930420K17Rik;4930471M23Rik;4930506M07Rik;4930547N16Rik;5031439G07Rik;5430427O19Rik;5430435G22Rik;6030451C04Rik;6330416G13Rik;9030425E11Rik;9030625A04Rik;9130014G24Rik;9230105E10Rik;9330175E14Rik;9530028C05;9530053H05Rik;A130071D04Rik;A630001G21Rik;A630033H20Rik;A630072M18Rik;A630081D01Rik;AA414768;AA467197;AA960436;AB124611;Abca1;Abcc1;Abcc3;Abcc5;Acer3;Acox3;Acp2;Acp5;Acsl1;Acsl4;Actn1;Acvrl1;Adam15;Adam17;Adam8;Adamtsl5;Adap2;Adar;Adcy7;Adhfe1;Adm;Adora2a;Adora2b;Adra1a;Adrb2;Adssl1;AF251705;Aff1;Afp;Aftph;Agtrap;Ahnak;Ahnak2;AI413582;AI429363;AI447881;AI467606;AI607873;AI662270;AI845619;Aif1;Aifm2;Aig1;Aim1;Aim2;Ak4;Ak8;Akap3;Akna;Akr1b8;Alas1;Alcam;Aldh1b1;Aldh3b1;Aldoc;Alox5ap;Amdhd2;Amz1;Ang;Angptl2;Ankrd33b;Ankrd37;Ankrd57;Anln;Anpep;Anxa1;Anxa3;Anxa4;Aoah;Ap1s2;Apaf1;Apbb1ip;Apobec1;Apobr;Apoe;Aqp9;Arap1;Arap3;Arf6;Arg1;Arg2;Arhgap10;Arhgap18;Arhgap19;Arhgap22;Arhgap25;Arhgap30;Arhgap4;Arhgap6;Arhgap9;Arhgef37;Arid3a;Arid5a;Arrb2;Arrdc4;Arsg;Asph;Ass1;Atf3;Atp10a;Atp13a2;Atp1a3;Atp6v0a1;Atp6v0d2;Atp6v1b2;Atp6v1c1;Atp8b4;Aurka;Aurkb;AW112010;Axl;B230120H23Rik;B3gnt2;B3gnt3;B3gnt8;B430306N03Rik;B4galt5;B4galt6;Bak1;Bambi_ps1;Batf;Batf2;Bax;BC006779;BC013712;BC028528;BC030867;BC046404;BC048355;Bcat2;Bcl10;Bcl2l1;Bcl2l11;Bcl3;Bcl6;Best1;Bex1;Bhlhe40;Bhlhe41;Bid;Birc3;Birc5;Blnk;Blvrb;Brca1;Brdt;Bst1;Bst2;Btg1;Btk;Bub1;Bzrap1;C030046G05;C130050O18Rik;C1qa;C1qb;C1qc;C3;C330027C09Rik;C3ar1;C530028O21Rik;C5ar1;C77080;C78513;Cadm1;Cadps;Calcrl;Camk1;Camk2d;Cap1;Capg;Car13;Car5b;Casp1;Casp4;Casp7;Cass4;Cbl;Cblb;Cbr2;Ccdc109b;Ccdc50;Ccdc86;Ccdc88b;Ccdc99;Ccl12;Ccl17;Ccl2;Ccl22;Ccl3;Ccl4;Ccl5;Ccl6;Ccl7;Ccl8;Ccl9;Ccna2;Ccnb1;Ccnb2;Ccnc;Ccnd1;Ccne2;Ccnf;Ccr1;Ccr2;Ccr3;Ccr5;Ccrl1;Ccrl2;Cd109;Cd14;Cd180;Cd200;Cd200r1;Cd200r4;Cd22;Cd274;Cd28;Cd300a;Cd300lb;Cd300ld;Cd300lf;Cd302;Cd33;Cd36;Cd37;Cd38;Cd40;Cd44;Cd48;Cd52;Cd53;Cd5l;Cd68;Cd69;Cd72;Cd74;Cd80;Cd83;Cd84;Cd86;Cd93;Cd97;Cd99;Cdc42ep2;Cdc6;Cdca2;Cdca3;Cdca5;Cdca8;Cdk1;Cdk14;Cdk18;Cdk20;Cdk6;Cdkn1a;Cdkn1c;Cdkn3;Cds1;Ceacam1;Cebpa;Cebpb;Cebpd;Cenpe;Cenph;Cenpi;Cenpk;Cenpn;Cenpp;Cenpw;Cep55;Cerk;Cfb;Cflar;Cfp;Ch25h;Chaf1b;Chek1;Chi3l3;Chst11;Chst7;Cited2;Ckap2;Ckap2l;Cks1b;Clcn7;Clec10a;Clec2i;Clec4a2;Clec4a3;Clec4b1;Clec4d;Clec4e;Clec4n;Clec5a;Clec7a;Clic4;Clic5;Cln8;Clspn;Cmklr1;Cmpk2;Cmtm3;Cndp2;Cnr2;Col18a1;Coro1a;Coro1c;Coro2a;Cotl1;Cox6a2;Cp;Cpa3;Cpd;Cpeb4;Creg1;Csf1;Csf1r;Csf2;Csf2ra;Csf2rb;Csf2rb2;Csf3;Csf3r;Csrnp1;Cst7;Cstb;Ctbs;Ctla2b;Ctnnd2;Ctsa;Ctsb;Ctsc;Ctsd;Ctsk;Ctsl;Ctss;Ctsz;Cttnbp2nl;Cx3cr1;Cxcl1;Cxcl10;Cxcl11;Cxcl13;Cxcl16;Cxcl2;Cxcl3;Cxcl5;Cxcl9;Cxcr3;Cxcr4;Cyba;Cybb;Cyp1b1;Cyp20a1;Cyp4v3;Cysltr1;Cyth4;D17H6S56E5;D2Ertd750e;D330040H18Rik;Dab2;Daglb;Daxx;Dcbld2;Dck;Dcp2;Ddhd1;Ddit3;Ddx58;Ddx6;Ddx60;Dennd1c;Dennd2a;Depdc1a;Depdc7;Dhrs3;Dhx58;Diap3;Dio2;Dlgap5;Dnahc2;Dnase1l1;Dner;Dnmt3a;Dock2;Dok1;Dok2;Dok3;Dpp7;Dppa3;Dram1;Dse;Dtx2;Dtx3l;Dtx4;Dusp1;Du

	sp13;Dusp2;Dusp23;Dusp28;Dusp3;Dusp4;Dusp5;E130306D19Rik;E2f7;E2f8;E330016A19Rik;E330022O07;Eaf1;Ebi3;Edem2;Edn1;Ednrb;Eepd1;Efcab4a;Efhd2;Egln3;Egr1;Egr2;Ehd1;Eif2ak2;Eif4e;Elmo1;Eme1;Emilin2;Emp2;Emp3;Emr1;Eno2;Enox2;Entpd1;Eps8;Epsti1;Erap1;Ercc6l;Ero1l;Esco2;Ets2;Etv5;Evi2a;Exo1;Expi;Ext1;Eya4;F10;F630043A04Rik;F630110N24Rik;F7;Fabp3;Fabp4;Fabp5;Fabp7;Fam114a1;Fam129b;Fam198b;Fam20c;Fam26f;Fam46c;Fam49a;Fam54a;Fam63a;Fam64a;Fam72a;Fam83f;Fas;Fblim1;Fbrs;Fbxo32;Fcer1g;Fcgr1;Fcgr2b;Fcgr3;Fcgr4;Fcna;Fcrl1;Fcrls;Fen1;Fermt3;Fes;Fgd2;Fgd4;Fgd6;Fgl2;Fgr;Fignl1;Fkbp15;Fkbp1b;Flrt2;Flrt3;Flt3l;Fn1;Fnip2;Folr2;Foxm1;Fpr1;Fpr2;Frmd4b;Fxyd5;Fyb;G530011O06Rik;Gadd45b;Galc;Galns;Galnt6;Galnt9;Gamt;Gas2l3;Gas7;Gatm;Gbp2;Gbp3;Gbp6;Gch1;Gcnt1;Gda;Gdf15;Gdf3;Gem;Gfi1;Ggta1;Gins1;Gins2;Gla;Glipr1;Glipr2;Glrp1;Glrx;Gm11110;Gm11428;Gm14005;Gm14446;Gm2a;Gm6377;Gm885;Gm9706;Gmfg;Gna13;Gna15;Gng2;Gngt2;Golph3l;Gpnmb;Gpr126;Gpr132;Gpr137b;Gpr137b_ps;Gpr18;Gpr183;Gpr35;Gpr65;Gpr68;Gpr84;Gpr85;Gprc5b;Gpsm3;Grap;Grn;Gsdmd;Gsr;Gsta3;Gtpbp2;Guca1a;Gusb;Gvin1;Gyk;Gys1;H2-Aa;H2_Ab1;H2_M3;H2_Q8;H2_T24;Hal;Hamp;Havcr2;Hbegf;Hck;Hcls1;Hcst;Hdc;Hebp1;Herc6;Hhex;Hif1a;Hip1;Hipk2;Hivep3;Hk3;Hlx;Hmga2;Hmga2_ps1;Hmha1;Hmmr;Hmox1;Hoxa1;Hp;Hpgds;Hpse;Hsd17b11;Hspa1a;Htr2b;Hvcn1;Icam1;Icosl;Ier2;Ier3;Ier5;Ifi203;Ifi204;Ifi27l2a;Ifi35;Ifi44;Ifi47;Ifih1;Ifit1;Ifit2;Ifit3;Ifitm6;Ifnar1;Ifnar2;Ifnb1;Ifng;Ifngr2;Ifrd1;Igf1;Igf2bp2;Igsf6;Igsf8;Igtp;Iigp1;Ikbkb;Ikbke;Ikbkg;Ikzf1;Il10;Il10ra;Il12a;Il12b;Il12rb2;Il13ra1;Il15;Il15ra;Il17ra;Il18;Il18bp;Il1a;Il1b;Il1f9;Il1rl1;Il1rn;Il20rb;Il21r;Il23a;Il2rg;Il3ra;Il4ra;Il6;Il7r;Incenp;Inhba;Inpp5d;Insl6;Ints12;Iqgap1;Iqgap3;Iqsec2;Irak2;Irak3;Irak4;Irf1;Irf5;Irf7;Irf8;Irg1;Irgm1;Irgm2;Isg20;Itga4;Itga5;Itga9;Itgal;Itgam;Itgax;Itgb2;Itgb7;Itgb8;Jak2;Jdp2;Junb;Kcnab2;Kcnk13;Kcnk6;Kcnn4;Kctd12b;Kdm6b;Kif11;Kif18b;Kif20a;Kif20b;Kif22;Kif23;Kif2c;Kitl;Klc4;Klf6;Klra2;Klrk1;Kpna3;Kpna4;Lair1;Lancl2;Laptm5;Lat;Lat2;Lck;Lcn2;Lcp1;Lcp2;Ldlr;Ldlrap1;Lfng;Lgals3;Lgals3bp;Lgals8;Lgals9;Lgmn;Lhfpl2;Lhx2;Lilrb3;Lipa;Lipg;Litaf;Lix1;Lmnb1;Lmnb2;Lmo2;Lnp;LOC545261;Lonrf3;Lox;Lpcat2;Lpl;Lpxn;Lrmp;Lrp1;Lrp11;Lrp12;Lrr1;Lrrc27;Lrrc33;Lrrc8c;Lsp1;Lst1;Ly75;Ly86;Ly9;Ly96;Lyl1;Lyn;Lyz1;Lyz2;Maf;Mafb;Maff;Malt1;Mamdc2;Man1c1;Map3k8;Map4k1;Mapk6;Mapkapk2;Marcks;Marcksl1;Marco;Mastl;Matk;Max;Mcl1;Mcm10;Mcm3;Mcm5;Mcoln2;Mcoln3;Mctp1;Mdm2;Mecp2;Mefv;Melk;Mertk;Met;Metrnl;Mfge8;Mfsd7b;Micall2;Mir155;Mitf;Mlkl;Mmp12;Mmp13;Mmp14;Mmp19;Mmp8;Mmp9;Mocos;Mocs1;Mospd2;Mov10;Mpa2l;Mpeg1;Mpst;Mr1;Mrc1;Mreg;Ms4a1;Ms4a4c;Ms4a6b;Ms4a6c;Ms4a6d;Ms4a7;Ms4a8a;Msn;Msr1;Mthfr;Mtmr11;Mtmr7;Mvd;Mx1;Mx2;Mxd1;Myadm;Mycl1;Myd88;Myo1e;Myo1f;Myo1g;Myo5a;Myof;Naglu;Naip2;Naip5;Naip6;Nampt;Nanos1;Nap1l2;Napepld;Napsa;Ncapg;Nceh1;Ncf1;Ncf2;Ncf4;Nckap1l;Ndc80;Nek2;Nek6;Nes;Neu1;Neurl2;Nfam1;Nfil3;Nfkb2;Nfkbia;Nfkbib;Nfkbid;Nfkbie;Nfkbiz;Nfya;Niacr1;Ninj1;Nlrc5;Nlrp3;Nme4;Nmi;Nod1;Nos2;Nostrin;Npc2;Nptx1;Npy;Nr1h3;Nrp2;Nt5c3;Nt5dc2;Nuf2;Nup54;Nupr1;Oas1c;Oas2;Oas3;Oasl1;Oasl2;Ogfr;Oit3;Olr1;Optn;Orai2;Orc1;Orm1;Osbpl3;Osgin2;Osm;P2rx4;P2rx7;P2ry1;P2ry14;P2ry2;P2ry6;P4ha1;Pacrg;Pacs2;Papss2;Parp12;Parp14;Parp9;Parvb;Parvg;Pbk;Pcdh7;Pcp4l1;Pctp;Pcyt1a;Pdcd6ip;Pde4b;Pde8a;Pde8b;Pdgfb;Pdlim4;Pdlim5;Pdpn;Pdxk;Peli1;Pf4;Pfkfb3;Pfkp;Phlda3;Phospho1;Pi4k2a;Pi4k2b;Pid1;Pif1;Pik3ap1;Pik3cd;Pik3cg;Pik3r5;Pik3r6;Pilra;Pilrb1;Pim1;Pion;Pkib;Pla1a;Pla2g15;Pla2g4a;Pla2g7;Plagl2;Plau;Plaur;Plbd2;Pld3;Pld4;Plek;Plekha2;Plekha4;Plekhf2;Plekhm1;Plekhm2;Plekhm3;Plekhn1;Plekho1;Plekho2;Plin2;Plk1;Plk2;Plk4;Plod1;Pltp;Plxdc1;Plxna1;Plxnc1;Pmf1;Pmp22;Pnp;Poc1a;Por;Pou2f2;Pou3f1;Pparg;Ppfia3;Ppfibp2;Ppm1h;Ppp1r15a;Ppp1r15b;Ppp1r3b;Prc1;Prdm1;Prelid2;Prg4;Prkch;Prkx;Procr;Prokr1;Prr11;Prss46;Psd4;Psen2;Psmb10;Psmb8;Psmb9;Psrc1;Pstpip1;Pstpip2;Ptchd1;Ptger2;Ptger4;Ptges;Ptgir;Ptgis;Ptgs2;Ptk2b;Ptplad2;Ptpn18;Ptpn22;

	Ptpn6;Ptprc;Ptpre;Ptprj;Ptpro;Ptrh1;Ptx3;Pvr;Pxn;Pydc4;Pyhin1;Qpct;Rab11fip1;Rab20;Rab31;Rab32;Rab39;Rab3il1;Rac2;Racgap1;Rad18;Rad51;Rad51ap1;Rad54l;Rai14;Ralgds;Rap1b;Rap2b;Rapgef6;Rasa4;Rasal3;Rasgef1b;Rassf4;Rassf8;Rbm47;Rbpj;Rbpms;Rel;Relb;Renbp;Retnla;Rgl1;Rgs1;Rgs14;Rgs16;Rgs18;Rhbdf2;Rhoc;Rhoh;Rhov;Rilpl2;Ripk2;Rnase4;Rnase6;Rnasel;Rnd1;Rnd3;Rnf128;Rnf149;Rnf180;Rnf19b;Rnf213;Rnh1;Rp2h;Rpa2;Rps6ka2;Rps6ka3;Rras;Rrm1;Rrm2;Rsad2;Rtp4;Rusc2;Rxra;Ryr1;S100a1;S100a4;S100a8;S100a9;S1pr2;Saa1;Saa3;Samd8;Samd9l;Samhd1;Samsn1;Sap30;Sash3;Sass6;Scarf1;Scgb1a1;Scpep1;Sdc1;Sdc3;Sdc4;Sdf2l1;Sel1l3;Selplg;Sema4c;Sema4d;Sepx1;Serpina3g;Serpinb2;Serpinb6b;Serpinb8;Serpine1;Sestd1;Sfpi1;Sgk3;Sgms2;Sgol1;Sgol2;Sgpl1;Sh2b2;Sh2d1b1;Sh2d3c;Sh3bgrl2;Sh3bp2;Sh3bp5;Sh3kbp1;Sh3pxd2b;Sh3tc1;Shcbp1;Shisa3;Siglec1;Siglece;Sirpa;Sirpb1a;Ska1;Skil;Skp2;Sla;Slamf1;Slamf7;Slamf8;Slamf9;Slc11a1;Slc11a2;Slc12a4;Slc13a3;Slc15a3;Slc16a10;Slc16a3;Slc22a4;Slc23a2;Slc24a6;Slc25a13;Slc25a24;Slc25a25;Slc25a30;Slc27a1;Slc29a3;Slc2a1;Slc2a6;Slc30a1;Slc31a1;Slc31a2;Slc36a4;Slc37a2;Slc39a14;Slc41a3;Slc43a2;Slc48a1;Slc6a12;Slc6a6;Slc6a8;Slc7a11;Slc7a2;Slc7a7;Slc7a8;Slc9a9;Slco3a1;Slfn1;Slfn10_ps;Slfn2;Slfn4;Slfn5;Slfn8;Slfn9;Slpi;Smc2;Smpdl3b;Smurf1;Snord89;Snrpd3;Snx10;Snx12;Snx20;Snx8;Soat1;Socs1;Socs3;Sod2;Sp100;Spag5;Speg;Sphk1;Spic;Spn;Spp1;Spred1;Spsb4;Src;Srd5a3;Srxn1;Ssh3;St18;St3gal1;St6galnac4;St8sia4;Stab1;Stac2;Stap1;Stard3;Stard5;Stat1;Stat2;Stat4;Stat5a;Steap3;Steap4;Stil;Stom;Stx11;Stx3;Susd3;Swap70;Syce2;Sykb;Syn1;Syngr1;Tal1;Tank;Tap2;Tapbp;Tapbpl;Tbc1d13;Tbc1d9;Tbxas1;Tceal7;Tcfec;Tcirg1;Tctex1d2;Tec;Tgfb1;Tgfbi;Tgif1;Tgm2;Thbs1;Tifa;Tifab;Timeless;Timp1;Tk1;Tle3;Tlr1;Tlr13;Tlr2;Tlr3;Tlr4;Tlr6;Tlr7;Tlr8;Tm6sf1;Tm7sf4;Tmcc3;Tmed5;Tmem104;Tmem106a;Tmem140;Tmem144;Tmem154;Tmem171;Tmem178;Tmem180;Tmem189;Tmem2;Tmem202;Tmem206;Tmem229b;Tmem26;Tmem67;Tmem71;Tmem82;Tmem86a;Tmigd1;Tmpo;Tnf;Tnfaip2;Tnfaip3;Tnfaip8;Tnfaip8l2;Tnfrsf11a;Tnfrsf13b;Tnfrsf1b;Tnfrsf22;Tnfrsf23;Tnfsf10;Tnfsf9;Tnip1;Tnnc1;Tnni2;Tor1aip1;Tor1aip2;Tor3a;Tox2;Tpcn2;Tpd52;Tpm3;Tpx2;Traf1;Traf3ip3;Traf6;Trafd1;Trem2;Trem3;Treml2;Trex1;Trib3;Trim13;Trim14;Trim21;Trim25;Trim30a;Trim30b;Trim30d;Trim34a;Trim36;Trip13;Troap;Trpm4;Trpv2;Tspan32;Tst;Ttc39b;Ttk;Ttyh2;Tubb6;Tusc1;Tyrobp;U90926;Uap1l1;Uba6;Uba7;Ubash3b;Ube2c;Ube2l6;Uchl1;Ugcg;Uhrf1;Ulbp1;Unc93b1;Upp1;Usp12;Usp18;Utp23;Vat1;Vav1;Vav3;Vcam1;Vcan;Vegfa;Vill;Vma21;Vnn3;Vrk2;Vsig4;Vwf;Wars;Was;Wdfy4;Wdr91;Wfs1;Wnk2;Xaf1;Xdh;Xist;Xkr8;Xylt2;Zbp1;Zc3h12a;Zc3h12c;Zc3h12d;Zc3hav1;Zdhhc2;Zeb2;Zfand5;Zfp36;Zfp799;Zfp811;Zmynd15;Znfx1;Zranb3;Zswim4;Zufsp;Zwilch.
Fibrosis Signature	*Acta2;Agt;Ccl1;Ccl12;Ccl3;Ctgf;Grem1;Il13;Il13ra2;Il4;Il5; Snai1; Col1a2; Col3a1; Lox; Mmp1a; Mmp13; Mmp14; Mmp2; Mmp3; Mmp8; Mmp9; Plat; Plau; Plg; Serpina1a; Serpine1; Serpinh1;Timp1; Timp2; Timp3; Timp4; Ccr2; Cxcr4; Ifng; Il10; Il13; Il13ra2; Il1a; Il1b; Il4; Il5; Ilk; Tnf; Bmp7; Cav1; Dcn; Eng; Inhbe; Ltbp1; Smad2; Smad3; Smad4; Smad6; Smad7; Tgfb1; Tgfb2; Tgfb3; Tgfbr1; Tgfbr2; Tgif1; Thbs1; Thbs2; Cebpb; Jun; Myc; Nfkb1; Sp1; Stat1; Stat6.*
GO: Retina Layer Formation	*Actl6a;Ahi1;Arl6;Atoh7;Atp2b1;Atp2b4;Atp8a2;Cabp4;Calb1;Casp2;Dscam;Fjx1;Foxn4;Gnat2;Gpm6a;Hcn1;Hipk1;Hipk2;Irx5;Lhx1;Megf11;Neurod1;Neurod4;Pde6c;Prom1;Psen1;Ptf1a;Ptn;Ptprm;Rab11fip4;Rdh13;Rom1;Rorb;Rp1;Rpe65;Rs1;Sdk1;Sdk2;Slc17a7;Slc17a8;Smarca4;Smarcd3;Sox8;Sox9;Tfap2a;Tfap2b;Thy1;Topors;Tspan12;Vsx1*
GO: Phototransduction	*Bca4;Accn1;Aipl1;Arrb1;Cabp4;Cds1;Cnga1;Cngb1;Gna11;Gnaq;Gnat1;Gnat2;Gnb1;Gngt1;Gngt2;Grk1;Guca1a;Guca1b;Nr2e3;Opn1lw;Opn1mw;Opn1mw2;Opn1sw;Opn3;Opn4;Opn5;Pdc;Pde6a;Pde6b;Pde6c;Pde6g;Pitpnm1;Plekhb1;Rcvrn;Rdh11;Rgr;Rho;Rp1;Rrh;Rs1;Sag;Trpc3;Unc119*

Supplementary table 2 – Antibodies

Antibody	Dilution	Species	Catalog#	Company
ClC-7	1:100 (IF)*<br>1:100 (IF)	Goat (C15)<br>Rabbit (H90)	sc-16444<br>sc-28755[¥]	Santa Cruz Biotechnology
Ostm1	1:100 (IF)	Goat (E12)	sc-168856[¥]	Santa Cruz Biotechnology
TGN46	1:1000 (IF)	Rabbit	ab16059	Abcam
Lamp1	1:200 (IF)	Mouse (H5G11)	sc-18821	Santa Cruz Biotechnology
γ-Adaptin	1:100 (IF)	Mouse (F10)	sc-398867	Santa Cruz Biotechnology
LC3b	1:500 (IF)<br>1:1000 (WB)	Rabbit	NB600-1384	Novus
F4/80	1:100 (IF)	Rat	MF48000	Invitrogen
ClC-3	1:100 (IF)	Rabbit (D8Y5Q)	133595[¥]	Cell Signaling
EEA1	1:100 (IF)	Rabbit (C45B10)	32885	
p-Smad2/3	1:200 (IF)<br>1:000 (WB)	Rabbit		Santa Cruz Biotechnology
SMAD2/3	1:200 (WB)	Mouse (C-8)	Sc-133098	Santa Cruz Biotechnology
GM130	1:100 (IF)	Mouse	sc-55591	Santa Cruz Biotechnology
GFAP	1:500 (IF)	Rabbit	Z0334	Dako
M6pr	1:1000 (IF)	Rabbit	ab-32815	Abcam
BiP1	1:1000 (WB)	Rabbit (C50B121)	31775	Cell Signaling
GRp94	1:200 (WB)	Rabbit (H212)	sc-11402	Santa Cruz Biotechnology
ERp57	1:200 (WB)	Rabbit (H220)	sc-28823	Santa Cruz Biotechnology
p62	1:1000 (WB)	Mouse	610833	BD Transduction Lab.
β-Actin	1:500 (WB)	Mouse (C4)	sc-47778	Santa Cruz Biotechnology

*IF: Immunofluorescence. WB: Western Blot.
[¥]: Validated

Supplementary table 3 – Statistical analysis by multiple comparison one way ANOVA(MC-ANOVA) of three-point data sets.

**Figure #**	**WT SCR vs. ADO2 SCR**	**WT SCR vs. ADO2 siRNA**	**ADO2 siRNA vs. ADO2 SCR**
	**p-Value**	**p-Value**	**p-Value**
7e	0.0017	0.2086	0.0246
7g	0.0001	0.1702	0.0015
7i	0.0015	0.5831	0.0042
8a	0.0009	0.4793	0.0018
8b	0.0027	0.3354	0.0131
8c	0.0133	0.8330	0.0089
8d	<0.0001	0.0020	0.0182
8e	0.0015	0.5831	0.0042
8f	0.0025	0.8558	0.0036
8g	<0.0001	0.4271	<0.0001
Suppl. 4g	0.9630	0.9972	0.9794

Supplementary table 4 – Fiji® software script

**Analysis**	**Fiji Script**
Co-Localization (Figures 3b-h/j-l, 4 j-l,8a-b and Supplemental Figure 4e-f).	selectWindow("Series002_z0_ch01.lif");<br>selectWindow("Series002_z0_ch02.lif");<br>setAutoThreshold("Default dark");<br>//run("Threshold...");<br>//setThreshold(44, 255);<br>setOption("BlackBackground", true);<br>run("Convert to Mask");<br>selectWindow("Series002_z0_ch01.lif");<br>setAutoThreshold("Default dark");<br>//run("Threshold...");<br>//setThreshold(29, 255);<br>run("Convert to Mask");<br>imageCalculator("AND create", "Series002_z0_ch01.lif","Series002_z0_ch02.lif");<br>selectWindow("Result of Series002_z0_ch01.lif");<br>run("Measure");<br>selectWindow("Series002_z0_ch01.lif");<br>run("Measure");<br>selectWindow("Series002_z0_ch02.lif");<br>run("Measure");
Ratio Image (Figure 8d and Supplemental Figure 4c)	run("Specify...", "width=512 height=512 x=256 y=256");<br>selectWindow("Series006_z0_ch01.tif");<br>run("Specify...", "width=512 height=512 x=256 y=256");<br>run("Subtract Background...", "rolling=50");<br>selectWindow("Series006_z0_ch02.tif");<br>run("Subtract Background...", "rolling=50");<br>run("32-bit");<br>selectWindow("Series006_z0_ch01.tif");<br>run("32-bit");<br>run("Smooth");<br>selectWindow("Series006_z0_ch02.tif");<br>run("Smooth");<br>setAutoThreshold("Default dark no-reset");<br>//run("Threshold...");<br>setAutoThreshold("Default dark no-reset");<br>run("NaN Background");<br>selectWindow("Series006_z0_ch01.tif");<br>setAutoThreshold("Default dark no-reset");<br>//run("Threshold...");<br>run("NaN Background");<br>run("Ratio Plus", "image1=Series006_z0_ch01.tif image2=Series006_z0_ch02.tif background1=0 clipping_value1=0 background2=0 clipping_value2=0 multiplication=1");<br>run("brgbcmyw");<br>//run("Brightness/Contrast...");

	run("RGB Color");<br>run("Save", "save=C:/Users/xxxx/xxxx/Ratio.tif");
Fluorescence intensity (Figures 3i,m, 4a-c, 6b-c, 8c/e-g and Supplemental Figure 4g)	run("Specify...", "width=512 height=512 x=256 y=256");<br>run("Measure");

Supplementary Table 5. Raw data of colocalization analysis.

Figure	Organelle's marker	Genotype	Total ClC7 pixel^2 (mean±SD)	Colocalized ClC7 pixel^2 (mean±SD)	Ratio (mean±SD)
**3b**	Calnexin	WT	58.06±34.68	20.13±5.76	0.34±0.14
		ADO2	53.14±38.19	23.86±8.07	0.45±0.17
**3c**	GM130	WT	95.98±39.86	52.82±30.54	0.55±0.20
		ADO2	130.75±79.77	107.32±67.83	0.82±0.08**
**3d**	TGN46	WT	76.95±51.86	29.13±28.31	0.37±0.13
		ADO2	70.32±38.94	46.63±31.86	0.66±0.23**
**3e**	γ-Adaptin	WT	43.88±20.97	1.94±1.16	0.04±0.007
		ADO2	59.10±14.96	22±2.97	0.37±0.04***
**3h**	Lamp1	WT	72.57±9.67	35.85±12.60	0.49±0.11
		ADO2	107.85±21.1	31.49±16.11	0.29±0.09*
**3j**	EEA1	WT	53.75±36.5	21.35±7.25	0.39±0.17
		ADO2	53.26±45.27	18.30±15.16	0.34±0.12
**3k**	ClC3	WT	87.1±57.23	48.72±21.16	0.55±0.23
		ADO2	95.65±66.27	28.13±22.56	0.29±0.11**
**3l**	M6PR	WT	73.6±7.72	17.69±4.58	0.24±0.05
		ADO2	69.42±28.71	16.63±4.64	0.23±0.16
**4j**	TGN46	WT	1612.4±508.7	367±182.11	0.23±0.06
		ADO2	2265.5±1177.4	772.01±320	0.34±0.05*
**4k**	γ-Adaptin	WT	3064.7±531.7	462.24±135.95	0.15±0.02
		ADO2	2168.5±465.84	597.90±212.12	0.27±0.08**
**4i**	Lamp1	WT	2837.3±738.3	708.36±189	0.25±0.07
		ADO2	1405.6±206.8	230.84±35.7	0.16±0.02*

*p≤0.05; **p≤0.01; ***p≤0.001

Supplementary table 6 – Primer pairs sequence

Primer pairs	Sequence
Tgfb1	Fw: CTGCTGCTTTCTCCCTCAAC<br>Rv: GACTGGCGAGCCTTAGTTTG
Tgfb2	Fw: GCTCCAATTCTTTCCCCTTC<br>Rv: CCCACCCATATGCTAACAAC
Tgfb3	Fw: CTGGGAGTCCTGAAGCTCAC<br>Rv: TGGTGCAAGTGGACAGAGAG
Acta2 (α-Sma)	Fw: CTGACAGAGGCACCACTGAA<br>Rv: CATCTCCAGAGTCCAGCACA
Clcn7	Fw: GCTGCTGCCTTTCAGTTGTC<br>Rv: TTCAAGAACTGCACCACTGC
Grem1	Fw: GACAAGGCTCAGCACAATGA<br>Rv: AACTTCTTGGGCTTGCAGAA
Col1α1	Fw: GTCCCTCTGGAAATGCTGGAC<br>Rv: GACCGGGAAGACCGACCA
Col3α1	Fw: GACCAGCAGTCCAACGTAGA<br>Rv: TCTCCAAATGGGATCTGTGG
Gapdh	Fw: TGGCAAAGTGGAGATTGTTGC<br>Rv: AAGATGGTGATGGGCTTCCCG
Ostm1	Fw: CCCAGTTACGGGAAAGATGA<br>Rv: TTACAACAAAGCCCGTTTCC
βActin	Fw: TGTTACCAACTGGGACGACA<br>Rv: TCTCAGCTGTGGTGGTGAAG
Hprt	Fw: CAGGCCAGACTTTGTTCCAT<br>Rv: TCCCCTCATCTTAGGCTTT

Supplemental Figures

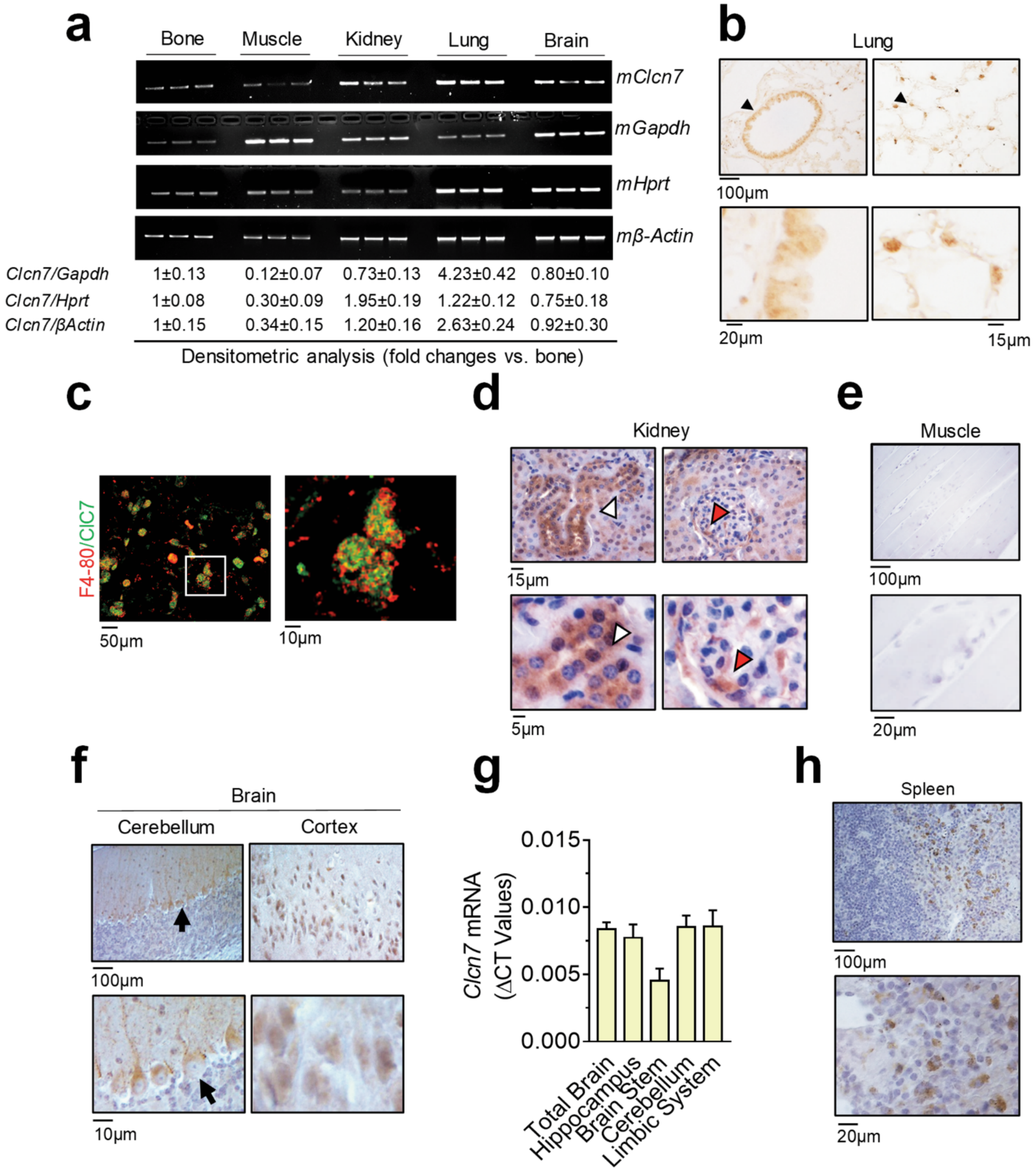

Supplemental Figure 1. Extra-skeletal *Clcn7* expression. (**a**) Semiquantitative RT-PCR for *Clcn7* in the indicated organs of 3-month-old wild-type C57BL6/J male mice. Gene expression was normalized using the indicated housekeeping genes. Lungs, kidneys, muscles, brains and spleens were harvested from 3-month-old wild type C57BL6/J male mice. (**b**) Immunohistochemistry analysis of ClC7 in the lung. Black arrowheads:

ClC7 positive cells (brown). (**c**) Immunofluorescence analysis of ClC7 (green) and the macrophages marker, F4/80 (red) in lung. (**d**) Immunohistochemistry analysis of ClC7 in kidney. White arrowheads: ClC7 positive cells (brown) in the tubular area. Red arrowheads: ClC7 positive cells in the glomeruli. (**e**) Immunohistochemistry analysis of ClC7 in muscle. (**f**) Immunohistochemistry analysis of ClC7 expression in the cerebellum. Black arrows: ClC7 positive Purkinje cells (brown). (**g**) Real time RT-PCR of *Clcn7* in the indicated brain regions. (**h**) Immunohistochemistry analysis of ClC7 in spleen. Images are representative, and data are the mean$\pm$SD of 3-5 mice per group.

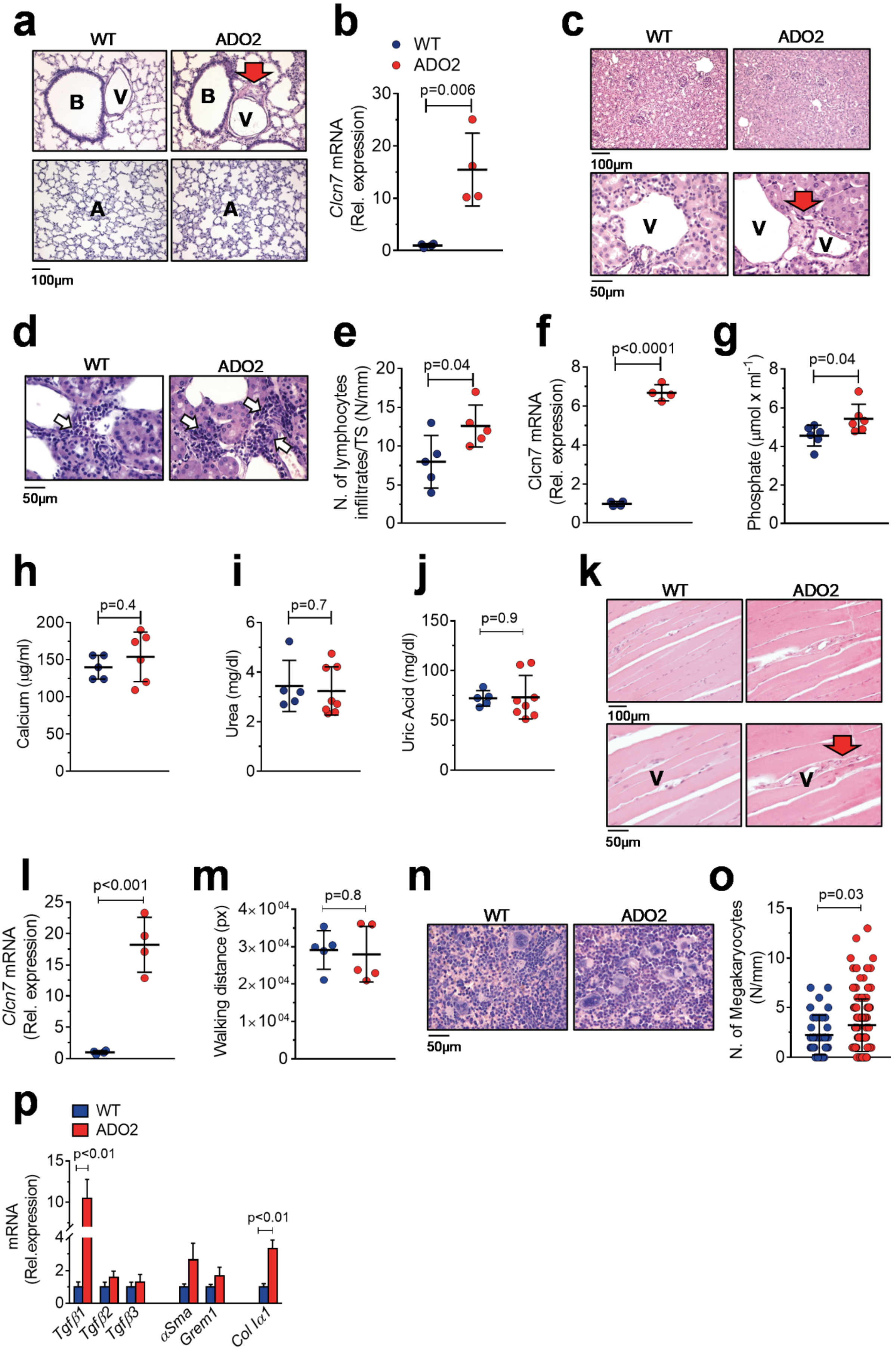

a
WT
ADO2
B
V
A
100µm
b
WT
ADO2
p=0.006
Clcn7 mRNA
(Rel. expression)
c
WT
ADO2
100µm
50µm
V
d
WT
ADO2
50µm
e
p=0.04
N. of lymphocytes infiltrates/TS (N/mm)
f
p<0.0001
Clcn7 mRNA
(Rel. expression)
g
p=0.04
Phosphate (µmol x ml-1)
h
p=0.4
Calcium (µg/ml)
i
p=0.7
Urea (mg/dl)
j
p=0.9
Uric Acid (mg/dl)
k
WT
ADO2
100µm
50µm
V
l
p<0.001
Clcn7 mRNA
(Rel. expression)
m
p=0.8
Walking distance (px)
n
WT
ADO2
50µm
o
p=0.03
N. of Megakaryocytes
(N/mm)
p
WT
ADO2
p<0.01
p<0.01
mRNA
(Rel.expression)
Tgfβ1
Tgfβ2
Tgfβ3
αSma
Grem1
Col Iα1

Supplemental Figure 2. Extra-skeletal phenotype. Lungs, muscles, kidneys, spleens and bone marrow were harvested from 12-month-old WT and ADO2 CD1 male mice. (**a**) Hematoxylin-eosin staining of lung sections. Red arrow: vessel wall thickening. B: bronchiole. V: vessel. A: alveoli. (**b**) Real time RT-PCR for *Clcn7* expression in WT and ADO2 lungs. (**c**) Hematoxylin-eosin staining of kidney sections. Red arrow: vessel wall thickening. V: vessel (**d**) Hematoxylin-eosin staining of kidney sections. White arrows: lymphocyte infiltrates. V: vessel. (**e**) Quantification of lymphocyte infiltrate numbers in kidneys. (**f**) Real time RT-PCR for *Clcn7* expression in kidneys. (**g**) Serum levels of phosphate, (**h**) calcium, (**i**) urea and (**j**) uric acid. (**k**) Hematoxylin-eosin staining of muscle sections. Red arrow: vessel wall thickening. V: vessel. (**l**) Real time RT-PCR for *Clcn7*. (**m**) Open Field (OF) test to measure the distance travelled by 3-month-old WT and ADO2 CD1 male mice. (**n**) Hematoxylin-eosin staining of spleen sections. (**o**) Quantification of the number of megakaryocytes. (**p**) Real time RT-PCR of the indicated genes in WT and ADO2 bone marrow. Images are representative, and data are the mean±S.D of 4-8 mice per group (Student's t test).

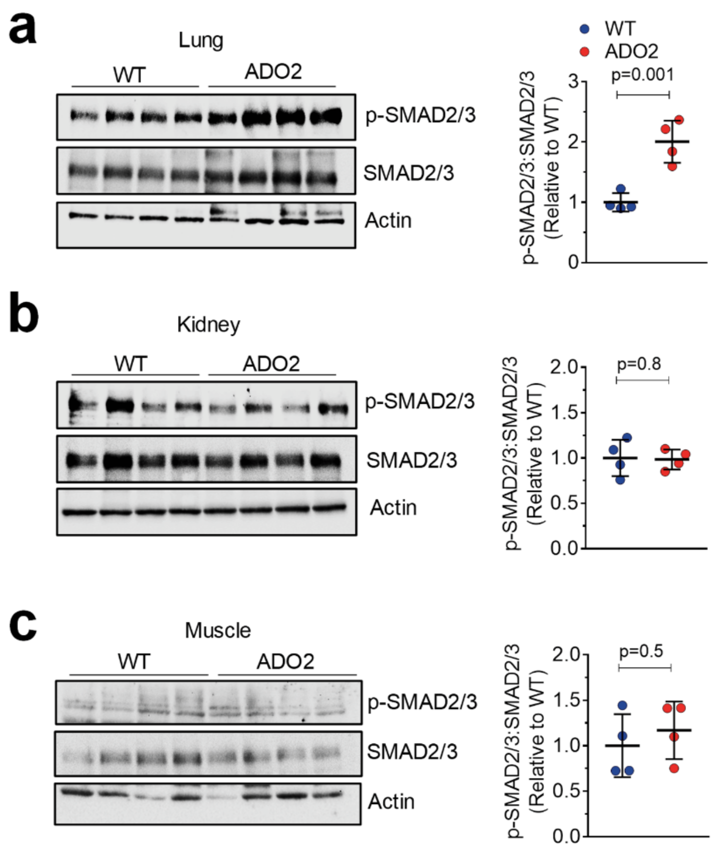

Supplemental Figure 3. Western blot analyses of SMAD2/3 phosphorylation. Lungs, muscles and kidneys were harvested from 12-month-old WT and ADO2 CD1 male mice. (**a-c**) Western blot analyses were performed for the indicated organs using specific antibodies for p-SMAD2/3 and SMAD2/3. Densitometric analyses were done using Image Lab® software (BioRad). Images are representative, and data are the mean ± SD of 4 animal/organ/group (Student's t test).

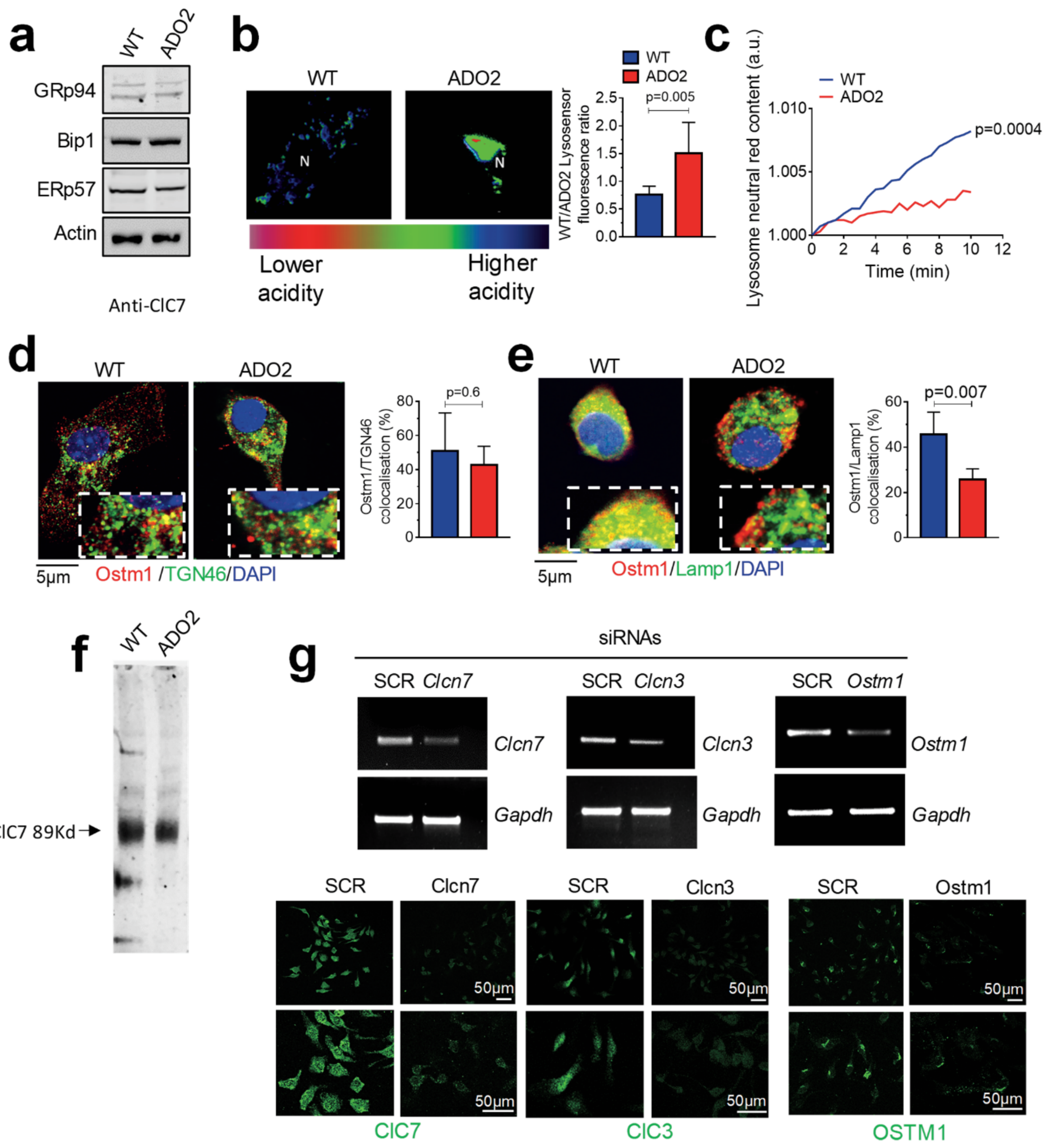

Supplemental Figure 4. Cellular phenotype. Primary BMMCs were isolated from 10-day old WT and ADO2 C57BL6/J mice. (**a**) Western bot analyses for the indicated ER stress proteins in WT and ADO2 BMMCs, normalized by actin. (**b**) Ratiometric analysis in Lysonsensor-loaded BMMCs. (**c**) Neutral red uptake measured by fluorometry at the indicated time points in lysosomes isolated from total bone marrow flushed out from long bones of 2-month-old WT and ADO2 mice. (**d**) Immunofluorescence analysis of OSTM1 (red) and the trans-Golgi marker, TGN46 (green), in WT and ADO2 BMMCs, and quantification of the OSTM1/TGN46 co-

localization. (**e**) Immunofluorescence analysis of OSTM1 (red) and the lysosome marker, Lamp1 (green), in WT and ADO2 BMMCs, and quantification of the OSTM1/Lamp1 co-localization. (**f**) In-house validation of the ClC7 antibody performed by Western blot. (g) In-house validation of ClC7, ClC3 and OSTM1 antibodies in BMMCs treated with scrambled (control) or *Clcn7*-, *Clcn3*- and *Ostm1*-siRNAs (upper panels) performed by immunofluorescence (lower panels). In (b,d,e) nuclei are stained in blue with DAPI. Images are representative, and graphs are the mean ± S.D of 3 independent experiments or 5 mice per group (Student's t test).

Figure 3b

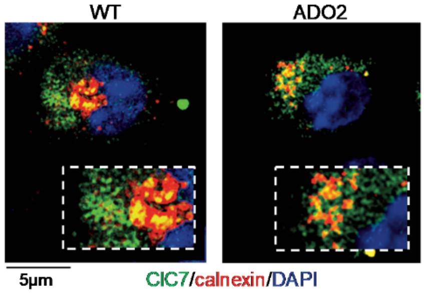

Figure 3c

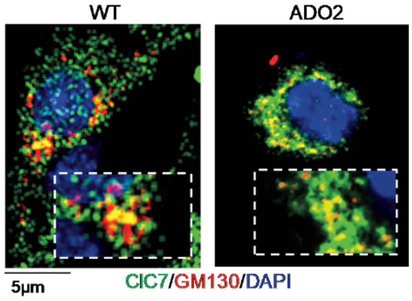

Figure 3d

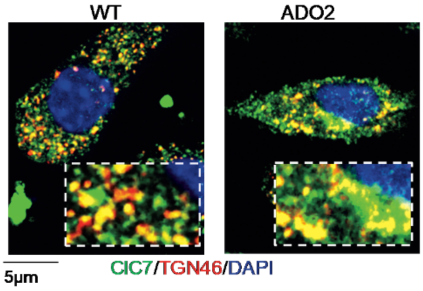

Figure 3e

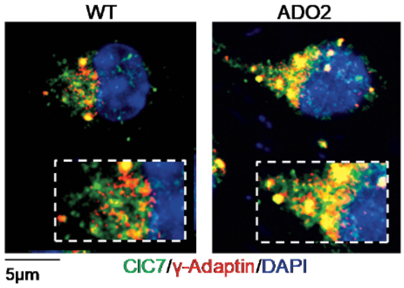

Figure 3g

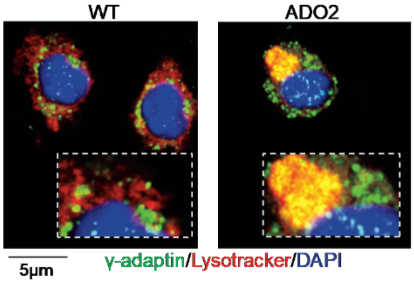

Figure 3h

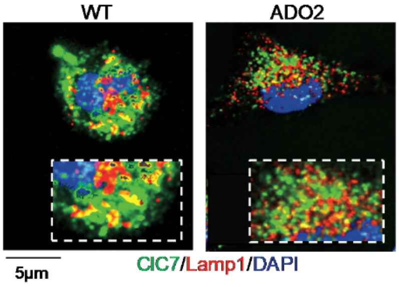

Figure 3j

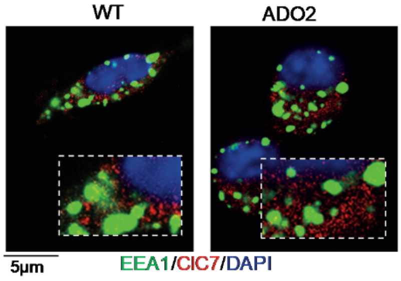

Figure 3k

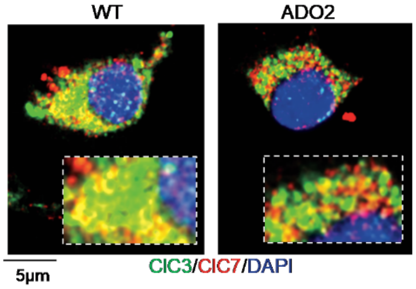

Figure 3l

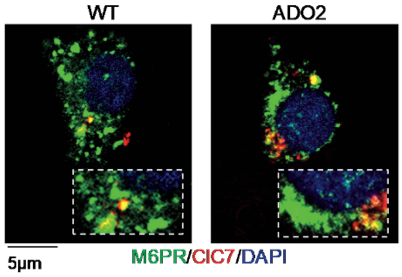

Supplemental Figure 5. Higher resolution and size of images displayed in Figure 3. The panels represent reproductions at higher resolution and size of the corresponding figure panels to facilitate the observation.

Supplemental references.

1. Capulli, M., Maurizi, A., Ventura, L., Rucci, N. & Teti, A. Effective Small Interfering RNA Therapy to Treat CLCN7-dependent Autosomal Dominant Osteopetrosis Type 2. *Mol. Ther. Nucleic Acids* **4**, e248 (2015).
2. Capulli, M. *et al.* The C-terminal domain of chondroadherin: a new regulator of osteoclast motility counteracting bone loss. *J. Bone Miner. Res.* **29**, 1833–1846 (2014).
3. David, D. J. *et al.* Neurogenesis-Dependent and -Independent Effects of Fluoxetine in an Animal Model of Anxiety/Depression. *Neuron* **62**, 479–493 (2009).
4. Arrant, A. E., Schramm-Sapyta, N. L. & Kuhn, C. M. Use of the light/dark test for anxiety in adult and adolescent male rats. *Behav. Brain Res.* **256**, 119–127 (2013).
5. Ennaceur, A. & Delacour, J. A new one-trial test for neurobiological studies of memory in rats. 1: Behavioral data. *Behav. Brain Res.* **31**, 47–59 (1988).
6. Vorhees, C. V & Williams, M. T. Morris water maze: procedures for assessing spatial and related forms of learning and memory. *Nat. Protoc.* **1**, 848–858 (2006).
